# Novel magnetic multicore nanoparticles designed for MPI and other biomedical applications: From synthesis to first *in vivo* studies

**DOI:** 10.1371/journal.pone.0190214

**Published:** 2018-01-04

**Authors:** Harald Kratz, Matthias Taupitz, Angela Ariza de Schellenberger, Olaf Kosch, Dietmar Eberbeck, Susanne Wagner, Lutz Trahms, Bernd Hamm, Jörg Schnorr

**Affiliations:** 1 Charité –Universitätsmedizin Berlin, Institute of Radiology, Berlin, Germany; 2 Physikalisch-Technische Bundesanstalt, Berlin, Germany; The University of Liverpool, UNITED KINGDOM

## Abstract

Synthesis of novel magnetic multicore particles (MCP) in the nano range, involves alkaline precipitation of iron(II) chloride in the presence of atmospheric oxygen. This step yields green rust, which is oxidized to obtain magnetic nanoparticles, which probably consist of a magnetite/maghemite mixed-phase. Final growth and annealing at 90°C in the presence of a large excess of carboxymethyl dextran gives MCP very promising magnetic properties for magnetic particle imaging (MPI), an emerging medical imaging modality, and magnetic resonance imaging (MRI). The magnetic nanoparticles are biocompatible and thus potential candidates for future biomedical applications such as cardiovascular imaging, sentinel lymph node mapping in cancer patients, and stem cell tracking. The new MCP that we introduce here have three times higher magnetic particle spectroscopy performance at lower and middle harmonics and five times higher MPS signal strength at higher harmonics compared with Resovist®. In addition, the new MCP have also an improved *in vivo* MPI performance compared to Resovist^®^, and we here report the first *in vivo* MPI investigation of this new generation of magnetic nanoparticles.

## Introduction

Having excellent magnetic properties and good biocompatibility, magnetic nanoparticles (MNP) based on magnetite have many technical and biomedical applications [1,2]. Technically, these MNP are used in data storage devices [3], for waste water treatment [4], or as catalysts or supports for catalysts in chemical processes [5,6]. In medical imaging, MNP have been used clinically as both T1 and T2 contrast agents for magnetic resonance imaging (MRI) [7–10]. Other researchers have shown that MNP are also suitable for therapeutic applications including hyperthermia for cancer treatment [11–15] and iron replacement therapy [16]. In regenerative medicine, MNP might be used for stem cell tracking with MRI [17–21]. Most MNP for biomedical applications are coated for colloidal stabilization during or after synthesis. Furthermore, MNP coatings can be functionalized with fluorescence dyes, antibodies, or proteins/peptides for bimodal detection of MNP or increased target specificity [22,23]. While MRI is well established, magnetic particle imaging (MPI) is a new emerging imaging modality. This fairly novel biomedical imaging modality is based on the nonlinear magnetization response of MNP to alternating magnetic fields [24] and can directly and specifically display MNP. Compared with MRI, MPI has very high temporal resolution, and a very good signal-to-noise ratio (SNR), allowing quantification of local MNP concentrations [25,26]. MPI can be combined with MRI [27,28] and appears to be particularly well suited for the spatially resolved visualization of rapid dynamic processes in real time such as the beating heart [29–32]. Other applications of MPI may include sentinel lymph node mapping in cancer patients [30,31], passive and active tumor targeting [33], and stem cell tracking [30,34–36]. Resovist^®^ is a liver-specific MRI contrast agent [7,37], that can be used as MPI Tracer and was taken off the market in Europe in 2008. Resovist^®^ has a bimodal magnetic size distribution and only the 30% fraction of larger magnetic cores with an equivalent core size of approx. 22 nm contributes significantly to the MPI signal [38]. Theoretical considerations indicate that single domain MNP with core sizes of about 25–30 nm are best suited for MPI and should be superior to Resovist^®^ [24,39,40]. Therefore, to further exploit the potential of this novel imaging modality, there is a need for improved MPI tracers [33]. The intensity of the MPI signal is dependent on the magnetic moment of the MNP used as tracers [33,38]. In a dispersion of MNP with high magnetic moments, the strong magnetic dipolar interaction between adjacent MNP may decrease colloidal stability. A possible approach to overcome this challenge is to synthesize clusters or so- called magnetic multicore particles (MCP). Because these clusters are composed of individual superparamagnetic cores, they might generate large magnetic moments in a magnetic field if there is sufficient ferromagnetic-like (parallel orientation of individual moments) interaction between single cores/crystals. On the other hand, in zero field the multicore structure might lead to higher colloidal stability in comparison to equivalent singlecore MNP because of the possibility of (partial) flux closure in zero field [38]. In addition, MNP dispersions for biomedical application need to be stable in physiologic media, biocompatible and biodegradable. Especially in vivo biodegradability has not yet been proven for any of the recently developed potential MPI tracers described in the literature [41–44]. Many different methods are available to synthesize iron-oxide-based MNP [1,45], and the most common are coprecipitation and thermal decomposition [1,33]. MNP synthesis using thermal decomposition results in pyrolytic decomposition byproducts of the basic materials due to radical reactions at high temperature(~300°C) that might hinder possible clinical application because of increased MNP toxicity [46,47]. Here we present a method for simple and reliable synthesis of stable aqueous dispersions of MCP with great potential for MPI, MRI, and other biomedical applications such as drug delivery or hyperthermia treatment.

## Results and discussion

### Nanoparticle synthesis and characterization

**Fig 1:**
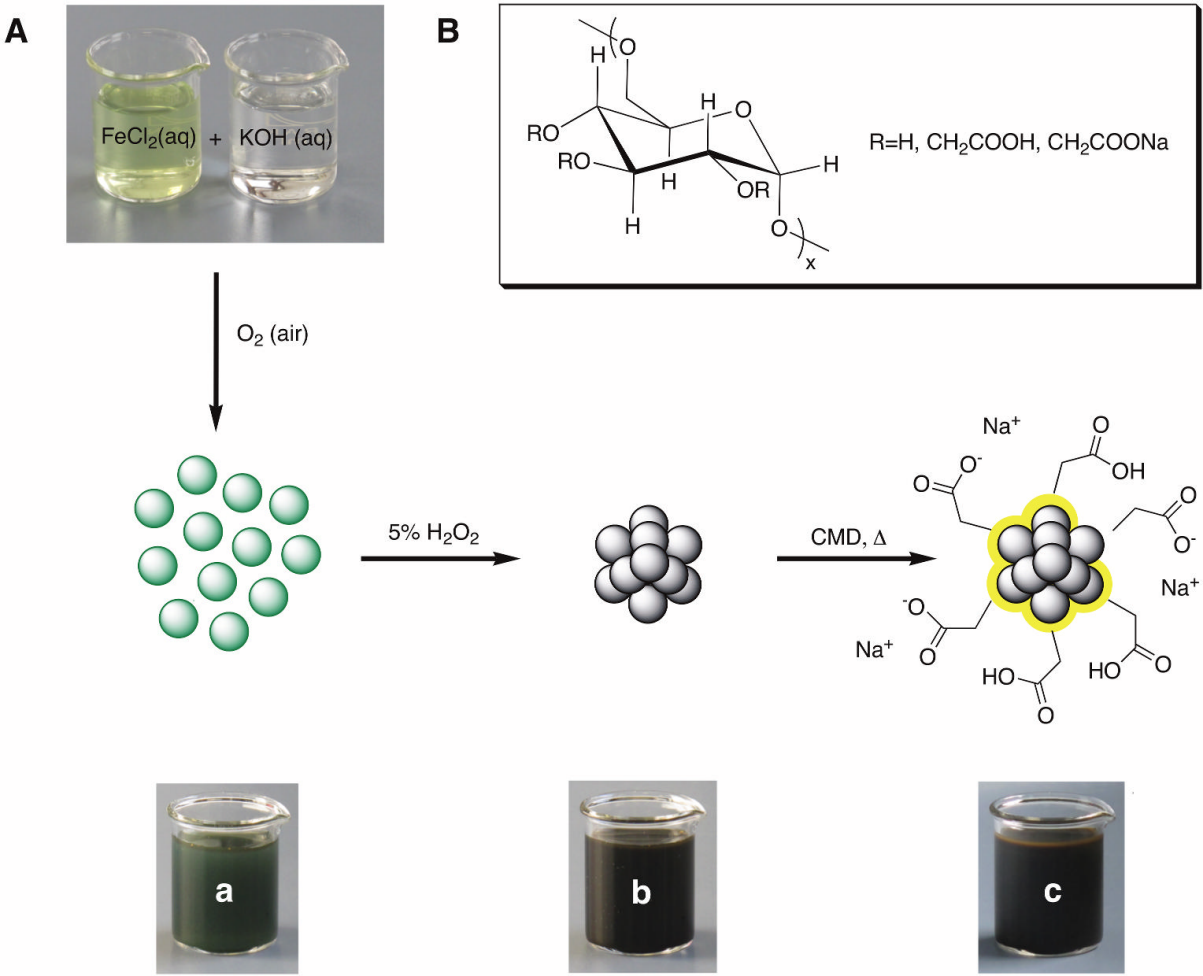
Nanoparticle synthesis. A) Magnetite synthesis with type 1 green rust as intermediate. a) Type 1 green rust—singlecore MNP. b) Fe_3_O_4_/γ-Fe_2_O_3_ -MCP. c) CMD-Fe_3_O_4_/γ-Fe_2_O_3_ -MCP. B) Carboxymethyl dextran sodium salt (CMD).

We chose a strongly modified coprecipitation method with relatively mild reaction conditions. Coprecipitation method has proven effective in the development of other iron oxide based drugs and contrast agents [7,20,37,48,49]. However, to obtain monodisperse MNP with high saturation magnetization using this procedure, considerable challenges had to be overcome [50–53]. Especially homogeneous reaction conditions, which are crucial for the formation of monodisperse MNP, are hard to achieve. All ingredients and chemicals that we used had proven biocompatibility and biodegradability and were used in the synthesis of approved medications or contrast agents before [45,54]. Unlike MNP synthesis based on thermal decomposition in organic solvents, the synthesis used here takes place in water and does not require the additional and complex step of phase transfer. The novelty of the developed synthesis presented here is the specific combination of individual steps. The first step of the synthesis method is to coprecipitate iron(II) chloride and KOH in the presence of atmospheric oxygen to obtain type 1 green rust [55–58] (Fig 1), to which hydrogen peroxide is added to yield Fe_3_O_4_ (magnetite) [59]. Because of the presence of oxygen during synthesis and storage, we assume the particles to consist of Fe_3_O_4_/γ-Fe_2_O_3_ (magnetite/maghemite) mixed-phase.

The synthesis parameters were chosen such that the pH of the dispersion following synthesis came close to the point of zero charge (PZC) of magnetite (pH = 6–6.8) [55] and maghemite (pH = 6.6) [60]. The MCP resulting after purification by magnetic separation were supplemented with a large excess of carboxymethyl dextran sodium salt (CMD) and then heated to 90°C for several hours. Heating, along with the large excess of CMD, aims at ensuring slow, controlled growth by aggregation or oriented attachment [61] of the MCP and partial reduction of γ-Fe_2_O_3_ to Fe_3_O_4_. In addition, long heating possibly improves the crystal structure of the MCP through annealing, since the ferrimagnetic properties of magnetite depend on the distribution of Fe^2+^/Fe^3+^ ions between A-sites and B-sites [62,63]. Another process that might improve the crystal structure is the simultaneous elimination of foreign ionic inclusions or imperfections of the crystal structure resulting from prior oriented attachment [61,64]. CMD coating of our MCP led to electrosteric stabilization, ensuring adequate stability of the MCP in aqueous dispersion at physiologic pH despite MCP large magnetic moments. The functional groups of the CMD coating allow chemical attachment of diverse molecules for further future biomedical applications [23]. The synthesis parameters during the development of MCP were systematically varied to iteratively optimize MCP in terms of signal intensity of the odd-numbered harmonics in magnetic particle spectroscopy (MPS). It turned out that a longer heating process after addition of CMD with probable simultaneous annealing and reduction combined with a larger amount of oxidizing agent had a strongly positive impact in the MPS/MPI characteristics. Another important point was the optimization of the magnetic fractionation of the particles under usage of a Base. The main difference between MCP 1 and MCP 2 is the quantity of oxidizing agent used during synthesis (Table 1).

**Table 1:** Difference between MCP 1 and MCP 2.

MCP	synthesis variant	annealing/ reduction (h)	oxidizing agent 5% H_2_O_2_ (ml)	magnetic separation
MCP 1	I	7,5	2	dispersion 2
MCP 2-1	II	8	3	dispersion 4
MCP 2-2	II	8	3	dispersion 5

**Fig 2:**
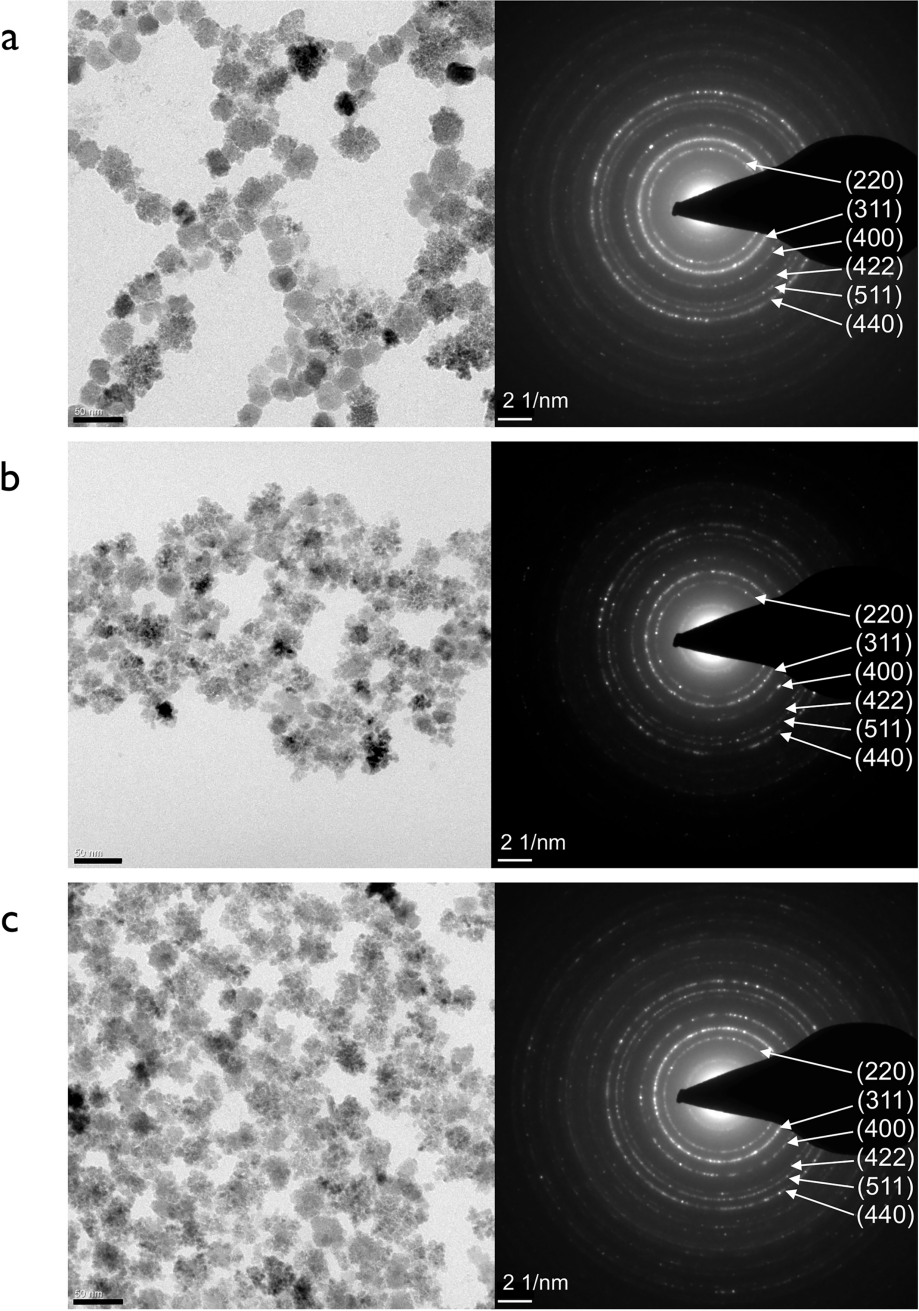
TEM images (scale bar: 50nm) on the left and corresponding SAED patterns (scale bar: 2 nm^-1^) on the right of (a) MCP 1, (b) MCP 2–1 and (c) MCP 2–2. For magnified TEM images of MCP 1 and MCP 2–2 see supplement.

Analysis of the three resulting MCP variants (MCP 1, MCP 2–1 and MCP 2–2) using high-resolution transmission electron microscopy (HRTEM) and the corresponding selected area electron diffraction (SAED) patterns (Fig 2A–2C) showed the MCP presumably to consist of magnetite with a predominantly clustered structure (multicore particles). For evidence of the simultaneous presence of maghemite for example x-ray diffraction (XRD) or Mössbauer investigations would be necessary [65,66].

**Fig 3:**
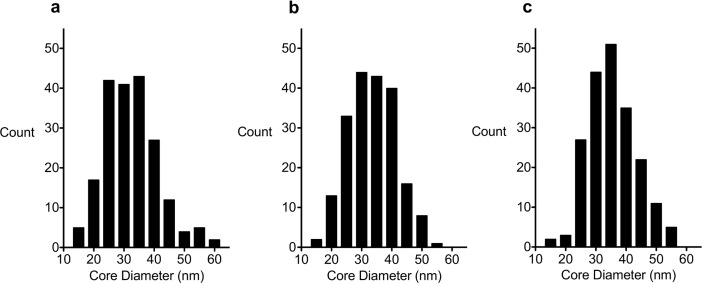
**TEM size distributions of (a) MCP 1, (b) MCP 2–1 and (c) MCP 2–2 based on measurement of 200 MCP in each case.** The y-axis of the histogram represents the number of particles.

While HRTEM showed that MCP 1 consisted of two types of MNP, MNP with a clustered structure (multicore particles) and others with an unknown structure (S1 Fig), the other two variants—MCP 2–1 and MCP 2–2 –consisted predominantly of MNP with a clustered structure (S1 Fig). Whereas MCP 1 consists of about 50% clustered particles, MCP 2–2 is composed of about 90% of these. Fig 3 presents the distribution of core sizes determined by TEM, and Table 2 lists the mean core sizes (d and d_V_) of the MCP along with other parameters. However, in case of the present MCP the effective domain size, i.e. the size of a domain with the same magnetic moment and the same saturation magnetization, is smaller than the physical size of the MCP. This relationship depends on many parameters like the single core size, its packing fraction, total MCP-size, as well as its inner structure. Another interesting question is, whether it is possible to separate the different kind of particles and do they equally contribute to the MPI signal? Possible methods for the separation are the field-flow fractionation (FFF) [67], or more precise the magnetic field-flow fractionation [68].

**Table 2:** Compilation of important properties of the MCP determined by magnetic measurement, TEM and DLS.

Particle	r1 [l mmol^−1^ s^−1^]	r2 [l mmol^−1^ s^−1^]	d TEM* [nm]	d_V_ DLS [nm] by volume	Z-Average [nm]	Pdi
MCP 1	16	300	32.53	24.4-58.8; m	55.0	0.172
MCP 2-1	20	350	33.56	24.4-58.8; m	47.2	0.074
MCP 2-2	17	404	35.31	28.2-68.1; m	52.4	0.083

* 200 MCP counted

To determine the potential of the MCP for MRI applications, we measured their relaxation rates (R1 and R2). The relaxivity coefficient r2, which is a measure of T2-weighted MRI contrast, is experimentally determined by calculating the relaxation rate (R2 = 1/T2) as a function of iron concentration. The spin-spin relaxation rate R2 is roughly proportional to the square of saturation magnetization (M_S_) [69,70]. The MCP have r2 values in the range of 300 to 404 l mmol^-1^s^-1^ (Table 2), which is very high for MCP synthesized from magnetite/maghemite in aqueous dispersion and suggests a high potential for T2- and T2*-weighted MRI applications.

**Fig 4:**
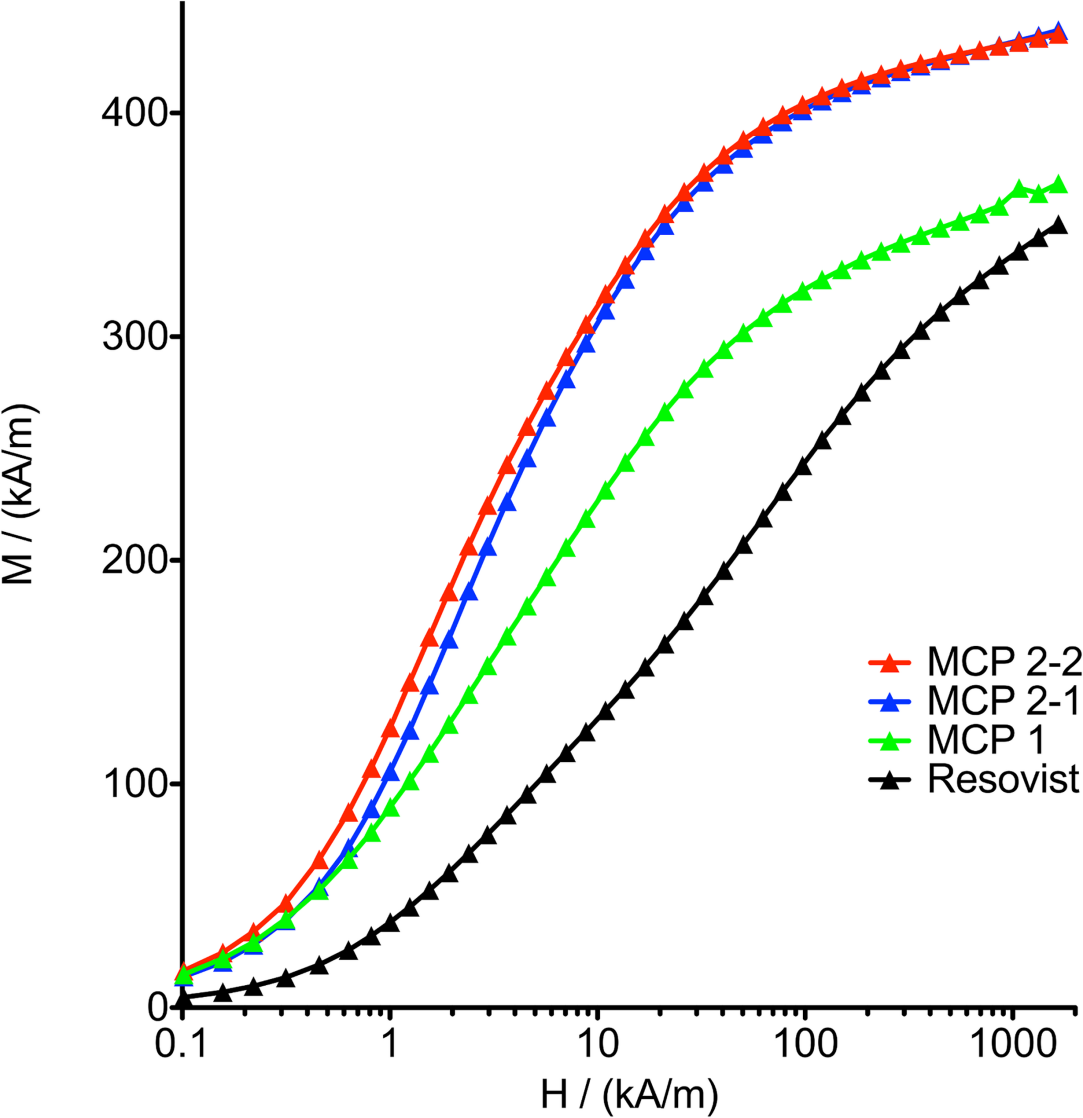
Mass magnetization *M* as a function of applied external field *H* measured for MCP 1, MCP2-1 and MCP2-2 with a SQUID at 295°C.

The MCP hydrodynamic sizes were measured by dynamic light scattering (DLS). In DLS measurements no aggregates could be detected (S2 Fig). The long-term stability of the MCP is also very good. In detail the particles dispersions of MCP 1 are stable at least for one year, MCP 2–1 and MCP 2–2 at least for a period of two years. In phosphate-buffered saline (PBS) the colloid is stable for at least 12 hours before aggregation arise. The synthesized MCP have M_S_ values of 95 to 115 Am^2^/kg Fe determined with a superconducting quantum interference device (SQUID) measuring M(H) at 295°K (Fig 4).

The M(H) curves were analyzed applying a model which describes the magnetization by the superposition of non-interacting MNP with different sizes as described in [38]. Using a lognormal distribution of the MNP diameters, the M(H)-data could not be described successfully. Thus we applied a bimodal lognormal distribution of the magnetic moments as it was found to be necessary also for Resovist [38]. The first mode comprises MNP with diameters smaller about 10 nm, which do not contribute to the MPS or MPI signal significantly [71]. Accordingly, in Table 3 only the parameters of the second, MPS-active mode, are listed.

**Table 3:** Fit parameters obtained from analysis of the M(H). Here *M*_s_ is the saturation magnetization while *β*, *d*_v2_, *σ*_2_, and *μ*_2_ denote the volume fraction, the diameter of mean volume, the dispersion parameter and the mean magnetic moment of the MNP of the second mode of the fitted bimodal lognormal distribution. *M*_3_ is the MPS-amplitude at 3^rd^ harmonics.

Sample	*β*	*d*_v2_ [nm]	*σ*_2_	*M*_s_ [Am^2^/kg Fe]	*μ*_*2*_ [aAm^2^]	*βμ*_*2*_ [aAm^2^]	*M*_3_ [Am^2^/mol(Fe)]
MCP 1	0.55	15	0.47	95	0.6	0.34	0.30
MCP 2–1	0.70	20	0.26	115	1.9	1.36	0.51
MCP 2–2	0.51	25	0.18	114	3.7	1.88	0.52
Resovist	0.23	24	0.21	98	2.7	0.61	0.20

Assuming a spherical shape of the particles, the distribution of effective magnetic diameters was derived from the magnetic moment distribution in order to get a comparison with diameters of the physical particle (TEM-data). The effective magnetic diameters of the mean magnetic moments or mean magnetic volume of the second mode, *d*_v2_, are clearly smaller than the mean physical (TEM-related) diameters. This is obviously attributed to the multicore structure of the MNP leading to a reduced moment in comparison to a singlecore MNP of the same size due to a packing fraction of the magnetic material smaller than one within the multicore MNP. Magnetic interaction among the single magnetic grains obviously creats a large main domain per multicore MNP which contributes to the second mode of the size distribution. This interaction seems to be of exchange nature, because the grains are crystallographically partially grown together (S3 Fig). On the other hand, within the less compact multicore MNP, there also remain smaller magnetic structures i.e. moments of single grains (one domain) or some correlated of them, which do not interact via exchange coupling. These structures seem to be attributed to the first mode of the obtained size distribution, comprising these smaller domains. In HRTEM we could find some indications for that hypothesis (S3 Fig). It seems that there are less dense areas in the multicore particles and sometimes different orientations of the crystal lattice are visible within one particle. Note, that these first hypotheses have to be checked by further investigations harnessing more methods for structure investigation like e.g. small and wide angle X-ray scattering (SAXS, WAXS). The most important parameter, derived from M(H)-data, which determines the MPS performance is the magnetic moment, here the mean of the second mode *μ*_2_. For MCP 2–2, MCP 2–1 and Resovist *βμ*_2_ correlates well with the MPS-amplitude at 3^rd^ harmonics *M*_3_ (Table 3). The deviation from this relation for MCP 1 might be attributed mainly to the much larger width of the size distribution *σ*_2_, making a proper comparison difficult because of the nonlinear relationship between moment and MPS signal.

In the literature, M_S_ bulk values of 111 and 127 Am^2^/kg Fe are reported for maghemite and magnetite, respectively [72,73]. Also in literature, aggregates, so-called Nanoflowers are described, which have a similar structure like our MCP in the TEM, but show only a saturation magnetization of about 60 Am^2^/kg Fe [74–76]. Actually, the reported saturation magnetization M_S_ of magnetite/maghemite nanoparticles is generally below pure bulk values [77–81]. This deviation from the bulk values of magnetite/maghemite is attributable to coordination effects of organic ligands [82,83] and/or a crystallographically disordered outer layer of MNP [84], often referred to a magnetic dead layer [85–87]. Hence, the with MCP obtained relatively high M_s_-values in the range of 110 Am^2^/kg or above seem to refer to a crystal structure close to that of pure magnetite, i.e. a low amount of crystallographic disorder.

**Fig 5:**
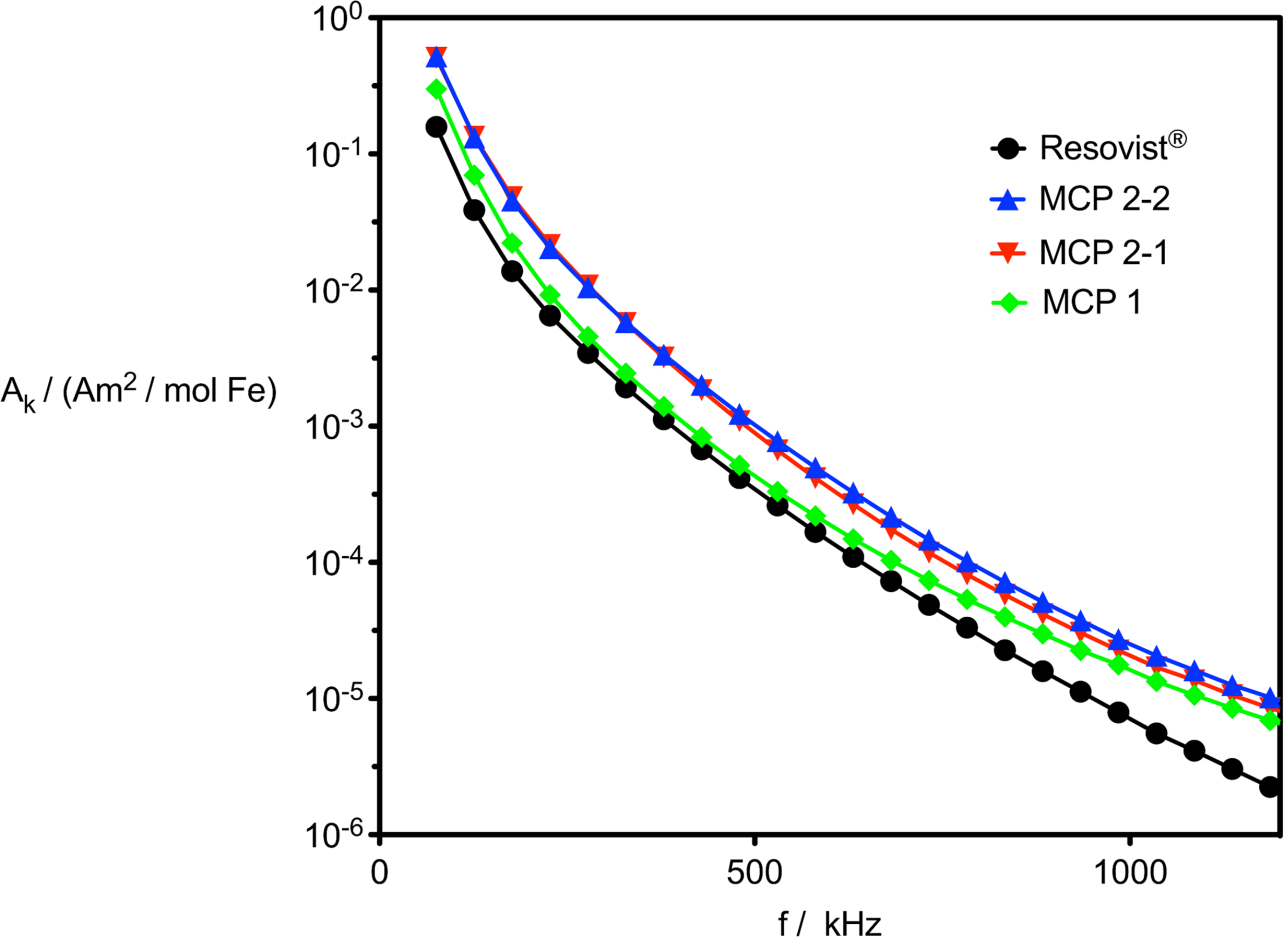
MPS data of MCP 1, MCP 2–1 and MCP 2–2 in comparison with Resovist^®^ (10 mT, 25kHz). Data are plotted as magnetic moment (normalized for iron content) versus frequency. Only odd harmonics are shown, and lines have been added to guide the eye.

With regard to MPS signal intensity, optimized MCP synthesis also resulted in MCP 2 with higher M_S_ and a lower polydispersity index (PDI) in comparison with MCP 1. The first step to test whether a new MNP is suitable for MPI is to investigate a specimen by MPS. In MPS, moments of the MNP are driven with a certain frequency in the kilohertz range (25 kHz in our case) when exposed to an alternating magnetic field. As a result of the MNP nonlinear magnetization response, higher harmonics of the basic frequency are generated, which are specific to the MNP and are measured inductively [24]. MPS can be regarded as a zero-dimensional type of MPI scanner without spatial resolution [88]. Resovist^®^ can be considered a gold standard in MPI—it is still the MPI tracer that has been mostly used in vivo [25,89,90] and was commercially available as contrast agent for clinical MRI in the past [91]. The MPS spectra obtained at 25 kHz and 10 mT show that the new MCP are superior to Resovist^®^ in terms of MPS performance (Fig 5). The signal strength of the MCP 1 sample is already superior to Resovist^®^ in the range of lower to middle harmonics of up to approx. 700 kHz, but its relevant superiority is seen at higher harmonics, where the MPS signal intensity of MCP 1 is four times stronger than that of Resovist^®^. The MPS signal intensities of lower and middle harmonics of MCP 2–1 and MCP 2–2 are three times higher and those at higher harmonics even five-times higher than those achieved by Resovist^®^.

### *In vitro* investigations

We compared the uptake of MCP 1 and Resovist^®^ by nonphagocytic cells (MSC) and phagocytic macrophages (RAW 264.7) using two protocols, with and without transfection agent (TA). Cationic TAs are commonly used to form positively charged NP complexes to facilitate cell membrane penetration of anionic MNP and increase their uptake by nonphagocytic cells such as MSC [92–95]. However, the use of TA with cells for human cell therapies will require additional FDA evaluation of the MNP-TA complex [96]. Therefore, alternative methods that avoid the use of TA are relevant for medical translation [21]. For this reason, we compared MCP 1 uptake with and without TA and in MSC. Although, phagocytic cells do not require protamine sulfate for MNP uptake, equal protocols were tested with RAW 264.7 macrophages for consistent comparison. Furthermore, the methodology used to achieve MNP-intracellular uptake was improved by removal of extracellular MNP. The quantification of the average intracellular MNP uptake was done by iron quantification and visualization by iron stain as previously published in our group [21]. In vitro biocompatibility of MCP 1 was tested after MNP uptake by mesenchymal stem cells (MSC) and macrophages (RAW 264.7) (S4 Fig and S5 Fig) for their effect on cell proliferation in comparison with unlabeled cells and cells labeled with Resovist^®^.

#### Mesenchymal stem cells

Overall, MSC were more efficiently labeled with MCP 1 than with Resovist^®^ with protamine sulfate as cationic transfection agent (TA) (0.2 mM: TA and 1 mM: TA) and without TA (0.2 mM). The use of TA increased the average intracellular uptake of MCP 1 (8- to 13-fold), resulting in average uptake of 10 pg Fe/cell (at 0.2 mM: TA; MNP loading concentration) and 15 pg Fe/cell (at 1 mM: TA; MNP loading concentration. Although the increase in MNP uptake with TA is higher for Resovist^®^ (16- to 26-fold with TA) than for MCP 1 (8- to 13-fold). The highest MSC uptake was achieved with MNP loading concentration of 1 mM: TA) with average MCP 1 uptake of 13 pg Fe/cell and average Resovist^®^ uptake of 9 pg Fe/cell (S4 Fig).

#### Macrophages (RAW 264.7)

Overall, this phagocytic cell line showed higher uptake of MCP than Resovist^®^ as confirmed by iron quantification after removal of extracellular iron (S5 Fig). Slight higher uptake of MCP 1 was observed with 0.2 mM MNP loading concentration without TA (~12 pg Fe/cell) than with TA (~8 pg Fe/cell). However, a higher MNP loading concentration (1mM) with TA significantly increased intracellular average MNP uptake by macrophages for both MCP 1 (up to 265 pg Fe/cell)) and Resovist^®^ (65 pg Fe/cell) (S5 Fig), As expected, overall uptake of MCP 1 and Resovist^®^ was higher in the phagocytic macrophage cell line (RAW 264.7) than in MSC. The uptake of MCP 1 was higher than that of Resovist^®^ independent of cell type and use of cationic TA. The negative charge of CMD coating of MCP 1 is stronger than that of carboxydextran coating of Resovist^®^. This might explain the higher affinity of MCP 1 to cationic TA and the higher cellular uptake of MCP 1 in comparison to Resovist^®^ as later discussed. In addition, MNP uptake increased with increasing MNP loading concentration with TA (S6 Fig, S7 Fig and S8 Fig).

We observed that larger complexes are formed by incubation of MCP 1 with TA than with Resovist^®^ in cell culture conditions (S6 Fig and S7 Fig). Larger MCP 1-TA complexes might be one reason for increased uptake for MCP 1-TA in comparison with the uptake for Resovist^®^-TA. Although, MCP 1 and Resovist^®^ are both sterically stabilized. Resovist^®^ is coated with carboxydextran and MCP 1 with carboxymethyl dextran (CMD). The additional CMD groups in MCP 1 accounts for their larger zeta potential (-32.8 mV) in comparison with Resovist^®^ (-25.1 mV). A larger surface charge of MCP 1 can increase the interaction with positive charged proteins such as fetal bovine serum (FBS) but also cell membrane components. These can cause that MCP 1-protein corona is formed, which can influence NP uptake. The addition of TA had a larger effect on uptake of MCP 1 for macrophages than for MSC (S4 Fig and S5 Fig) (FACTORS correspondently). Suggesting two different mechanisms for MCP 1-TA uptake in MSC and macrophages. Considering that the formation of a MNP protein corona not only influences uptake by cells but MNP stability [97]. We observed that the well plates where cells were incubated without TA, remained free of MNP-TA aggregates (S6 Fig: e—h for MSC and S7 Fig: e—h for macrophages), suggesting better MCP 1 stability when TA was avoid. In addition, MCP 1 uptake was increased in both cells by increasing MNP loading concentration to 1mM. Although the cellular mechanisms for MCP 1 uptake are beyond the scope of this manuscript. We speculate an endocytosis-independent pathway by diffusion for the internalization of MCP 1-TA (S6 Fig and S7 Fig). Future investigations will be required to prove this theory by comparing MCP 1-TA uptake in presence of inhibitors for the endocytic pathways [98]. Overall, the protocol for cellular uptake of MCP 1, is improved by increasing MCP 1 loading concentration to 1 mM, elimination of the use of TA, and inclusion of ECM digestion to remove extracellular MCP 1. This methodology provides an improved protocol for intracellular labeling of MSC with MCP 1 and Resovist [21] and macrophages with MCP 1 as shown in this manuscript. The reduction of extracellular MCP 1 by ECM digestion and cell passage is exemplarily shown for MCP 1 at 0.2 mM and 1mM loading concentrations in supplementary S8 Fig (S8 Fig).

#### Effect of MCP on the proliferation of MSC and macrophages

The effect of MCP 1 on MSC proliferation was tested over 12 days by measurement of population doubling time (PDT) of MCP 1-labeled cells and compared with the PDT of Resovist^®^-labeled and unlabeled cells. Despite the higher uptake of MCP 1 in comparison with Resovist^®^, the PDT of both MSC and macrophages was similar for cells labeled with MCP 1 and Resovist^®^ in comparison with unlabeled cells (S9 Fig). Additional studies published by our group confirm that MCP efficiently label MSC, enabling MRI with single cell sensitivity. Furthermore, MCP -labeled MSC maintained their in vitro stem-cell-like character, and features such as colony-forming unit capacity, in vitro multilineage differentiation capacity (adipogenesis, chondrogenesis and osteogenesis), and expression of MSC surface markers (CD90, CD44, CD73 and CD133) remained unmodified [21]. These findings further support the in vitro biocompatibility of MCP with MSC. Further experiments such as migration assays for MSC and investigations to test the immune responsiveness of macrophages should be performed with a view to specific biomedical applications of MCP 1-labeled cells. In addition, longitudinal measurements of MRI but also MPS and MPI signal will be required to confirm in vivo the stability of MNP in intracellular compartments. Two possible applications of MCP deserve special mention. First the uptake of MCP 1 by MSC is increased in comparison with Resovist^®^ and their detectability by MPI should be further explored. Second, higher uptake of MCP 1 than Resovist^®^ by macrophages should be carefully evaluated for applications that include intravenous systemic application. However, good uptake of MCP 1 by macrophages could in the future be exploited for specific imaging and theranostic targeting of macrophage-associated diseases [99], and these applications should also be explored for new generation MPI-MNP such as MCP 1.

### First *in vivo* studies and MRI experiments

**Fig 6:**
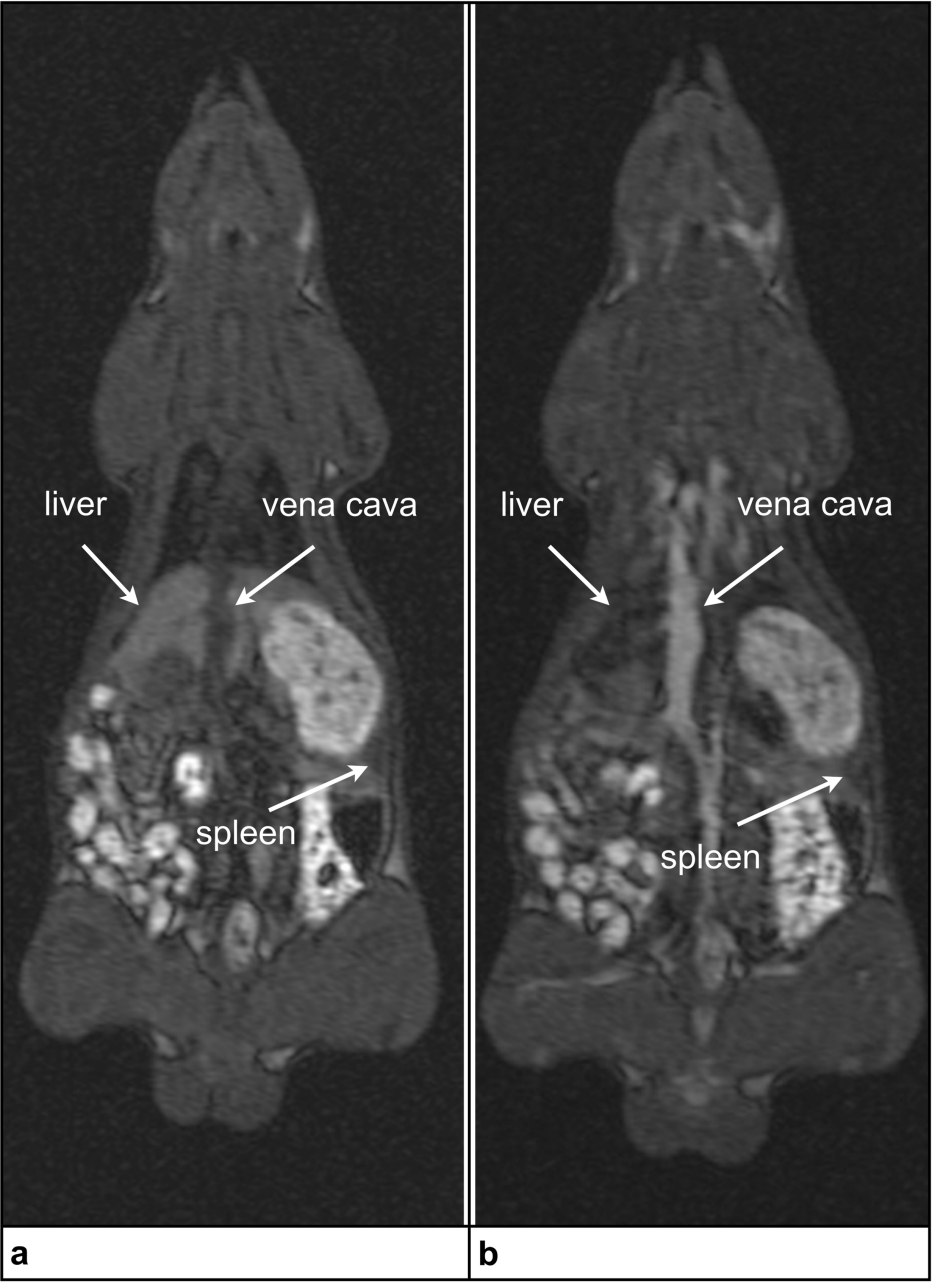
*In vivo* MRI at 1.5 Tesla: T1-weighted 3D gradient-echo (GRE) fast low angle shot sequence. a–Image without administration of MCP 1; b–Image obtained 2 minutes after administration of 0.1 mmol Fe/kg of MCP 1.

**Fig 7:**
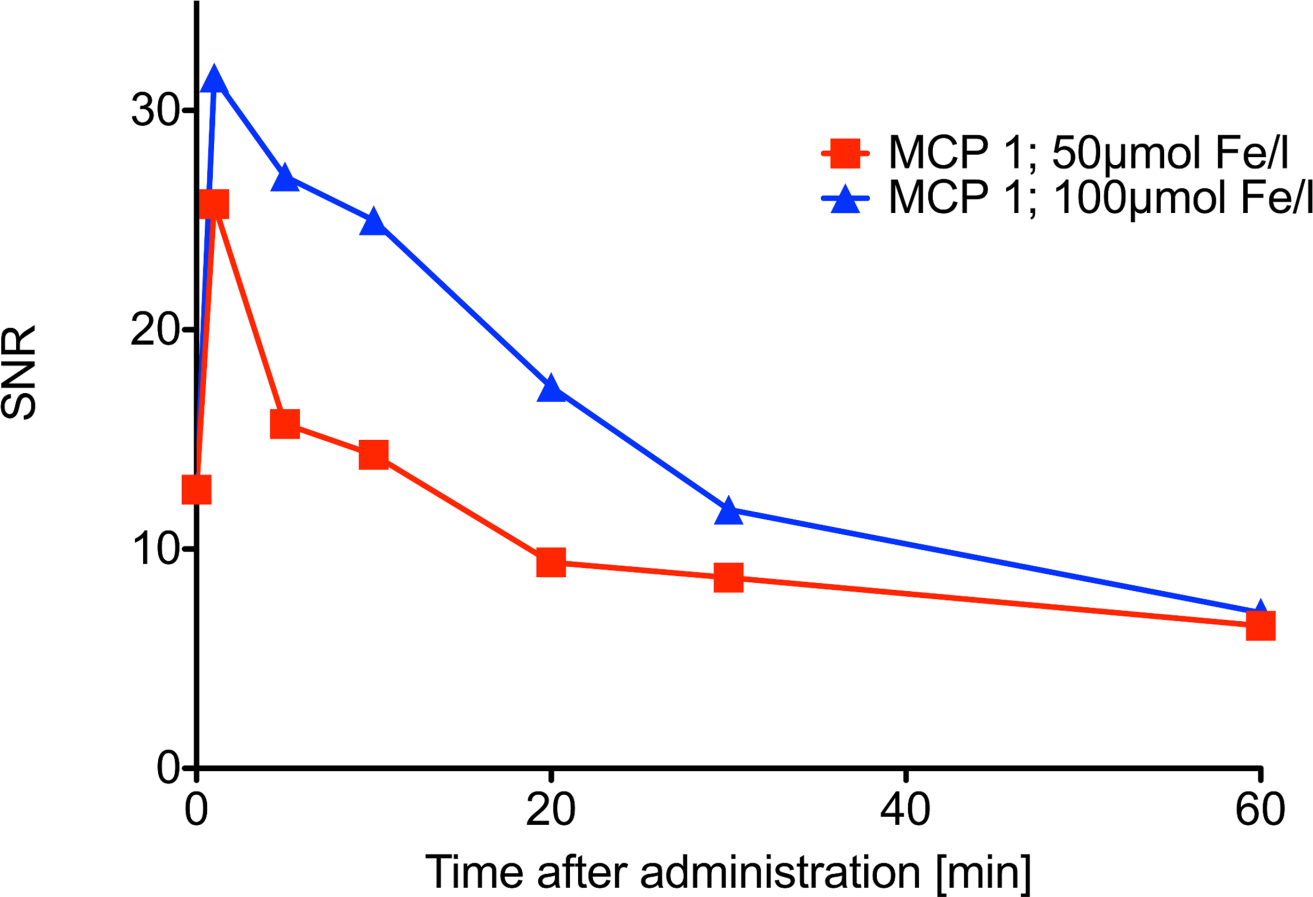
T1 weighted MRI blood half-life measurement of MCP 1 (50 and 100μmol Fe/kg) at 1.5 Tesla: T1-weighted 3D gradient-echo (GRE) fast low angle shot sequence.

MRI was used to determine the blood half-life of MCP 1 in rats. A total of 6 Sprague Dawley^®^ rats (SD rats, Charles River, Sulzfeld, Germany) were examined by T1-weighted and T2*-weighted MRI. The effect of MCP 1 was a transient signal enhancement within the vasculature in T1 weighted MRI and a signal decrease of the liver parenchyma in T1 and T2 weighted MRI, due to the well-known uptake of nanoparticles by phagocytic cells in the liver. The T1 weighted MRI blood half-life of MCP 1 was 8.8 and 17.4 min at 50 and 100 μmol Fe/kg, respectively, as measured using serial T1 weighted MRI (Figs 6 and 7).

Duration of degradation of MCP 1 in the liver, as measured using T2*-weighted MRI, was 5 weeks with a half-life of 7 days. Please note that MRI is not quantitative. A total of 4 Spraque Dawley (SD) rats (Charles River, Sulzfeld, Germany) were examined for *in vivo* compatibility. MCP 1 *in vivo* compatibility was studied using doses of up to 3 mmol Fe/kg of body weight, and overall no adverse effects such as reduced motility or piloerection were observed.

#### Initial *in vivo* MPI experiments

**Fig 8:**
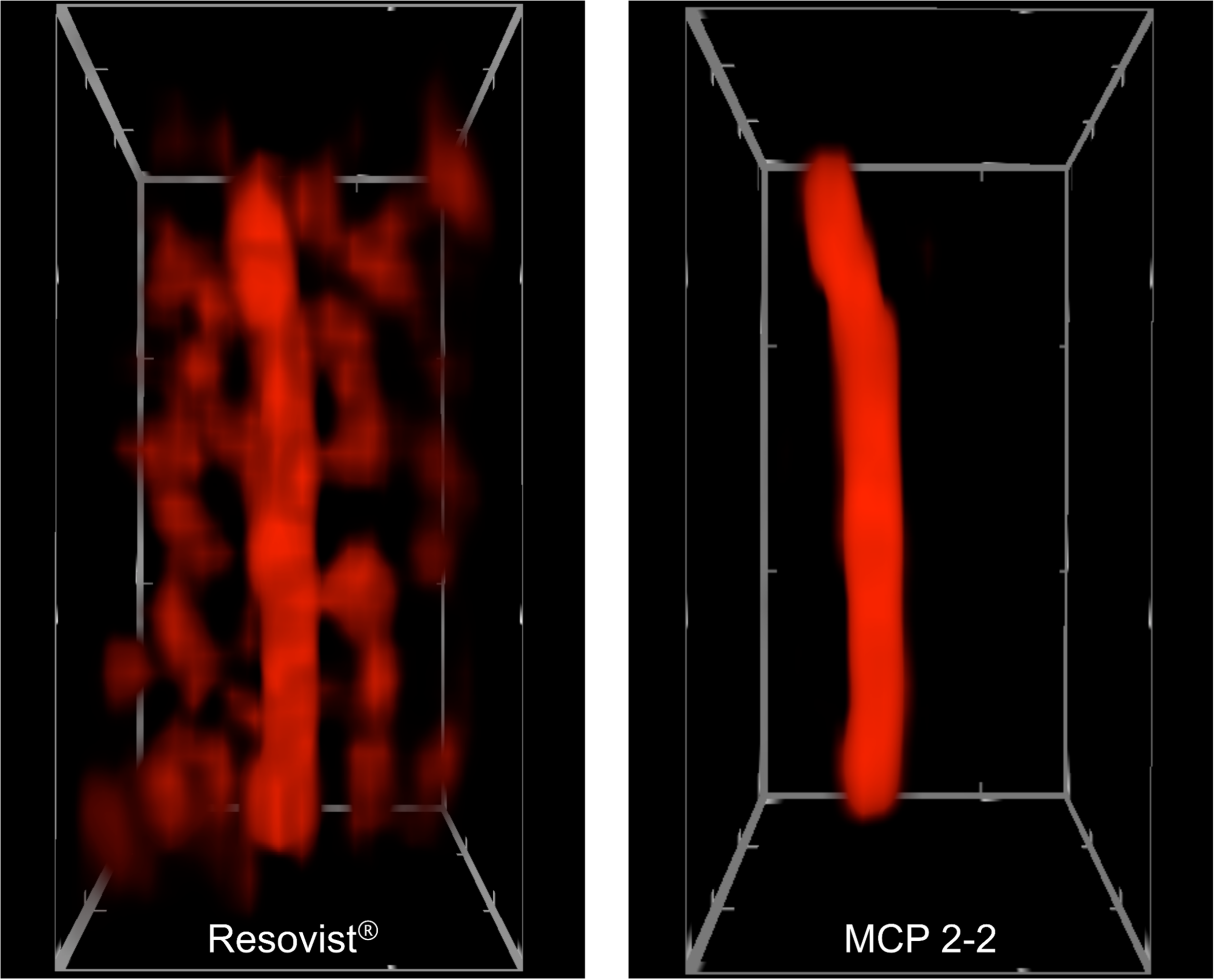
*In vivo* MPI image of vena cava of a rat after i.v. bolus administration of 0.05 mmol Fe/kg (0.1 ml) Resovist (left) and MCP 2–2 (right) respectively. For both image reconstructions the same parameters were used. 3D volume of field of view (FOV) with a size of 28 x 28 x 14 mm^3^ is shown.

**Fig 9:**
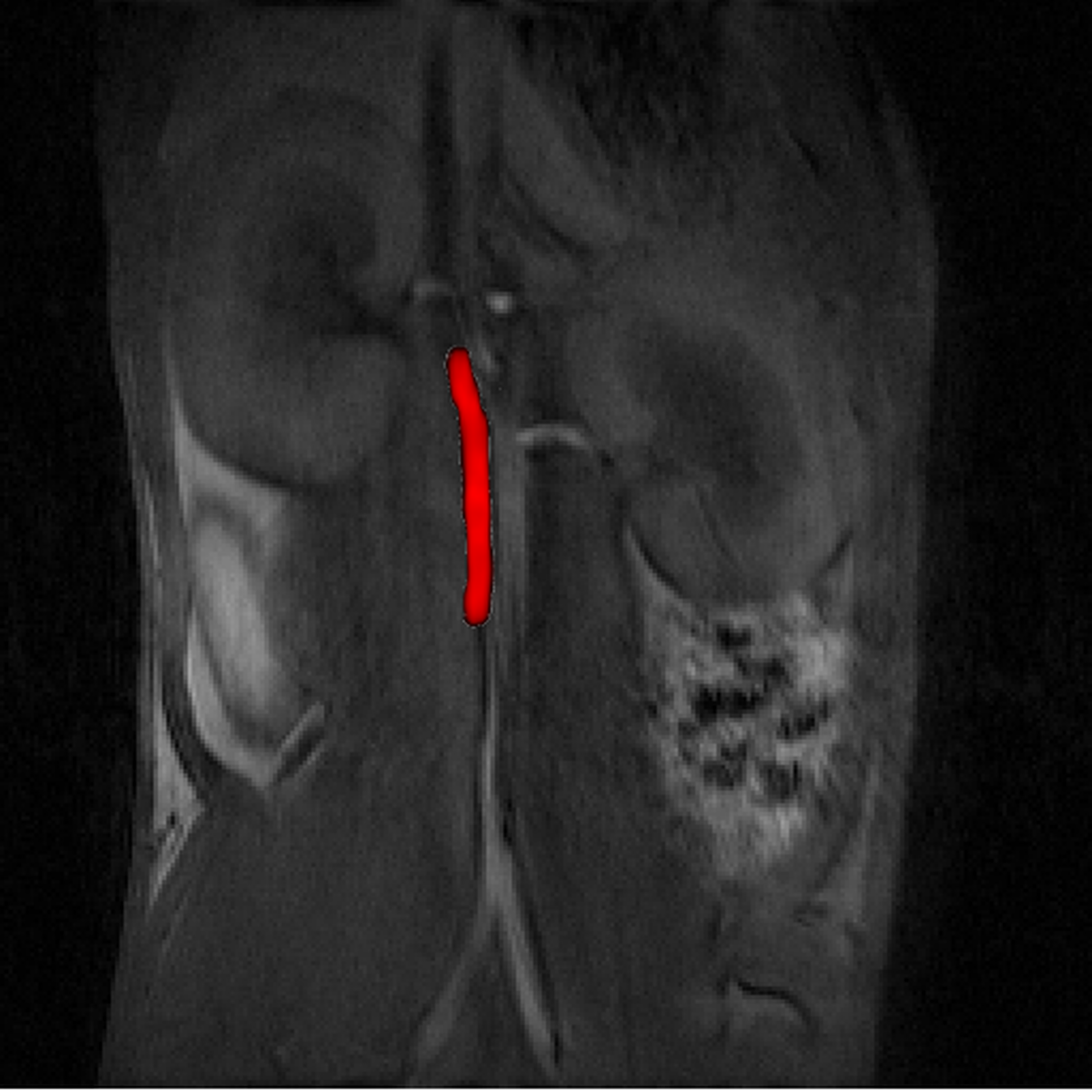
*In vivo* MRI image at 1.0 Tesla (ICON MRI, Bruker): T1-weighted 2D gradient-echo (GRE) fast low angle shot sequence overlaid with 3D volume *in vivo* MPI image of Fig 8 of the same rat.

To study the MPI behavior of MCP 2–2 in a preclinical MPI scanner (Bruker Biospin GmbH, Ettlingen, Germany) [28], a total of 2 SD rats (Charles River, Sulzfeld, Germany) were examined using Resovist^®^ and MCP 2–2 with doses of 0.1 mmol Fe/kg and 0.05 mmol Fe/kg, respectively. These in vivo experiments showed high intravascular MPI signal intensities, allowing adequate evaluation of the images. These initial experiments also showed MCP 2–2 to have good imaging properties. A direct comparison of *in vivo* MPI data of MCP 2–2 and Resovist^®^ using the same reconstruction parameters revealed the superiority of our newly developed MPI-Tracer (MCP 2–2) over Resovist^®^ in terms of higher S/N and better anatomical delineation of the blood vessel (Figs 8 and 9). A method for accurate co-registration of MPI and MRI data is currently being developed. Another interesting aspect would be the comparison with LS-008 tracer (LodeSpin, Seattle, USA). These Particles showed a mean amplification of amplitudes of 3.4 compared to Resovist in MPS at 14 mT and 25 kHz and a better spatial MPI resolution [100]. Because of its polyethylene glycol (PEG) coating LS-008 shows a long circulation time and is used as a blood pool tracer [44,100].

## Conclusions and outlook

In summary, we present a novel aqueous synthesis for generating MCP with excellent magnetic characteristics, and therefore highly suited for both MRI and MPI, and could allow the combination of these two techniques for bimodal imaging. The innovation of the new synthesis lies in the oxidation of green rust to a probably magnetite/maghemite mixed-phase in conjunction with subsequent annealing and parallel partly reduction at 90°C for several hours. Our results indicate, that MCP containing aggregates composed of uniform small single crystals lead to improved MPI performance. Experimental MPS and *in vivo* MPI data demonstrate the superior performance of MCP in comparison with Resovist^®^. In addition we show that MCP 1 is in vitro biocompatible after intracellular uptake and can be used to efficiently label cells to further study their potential for in vivo cell tracking by MRI and MPI in regenerative medicine and stem cell therapies. Furthermore, MCP 1 did not have in vivo adverse effects at doses of up to 3 mmol Fe/kg body weight, and showed a liver half-life of about 7 days. Because MCP 1 and MCP 2 have the same CMD coating and the same basic core structure, we assume that these MNP also have similar in vitro and in vivo characteristics. Nevertheless, further investigations are required to test the in vitro and in vivo properties of MCP 2 in more detail. The presented initial MPI experiments for imaging the vena cava of rats are the first in vivo MPI studies using MCP 2–2 as improved MPI tracers of a new generation synthesized by coprecipitation. The next challenge is to show that the new MPI tracers are suitable for cardiovascular imaging and further biomedical applications such as sentinel lymph node mapping in animal models of cancer or stem cell tracking. Further efforts will aim at optimizing the pharmaceutical formulation of MCP and increase specificity by functionalization of the MCP coating, e.g., with antibodies for targeted imaging or with drugs for specific uses as theranostics.

## Experimental section

### Materials and instruments

All chemicals were purchased from Sigma-Aldrich (Steinheim, Germany). Iron(II) chloride tetrahydrate, carboxymethyl dextran sodium salt and potassium hydroxide were used as received. To prepare 5% hydrogen peroxide solution (5 wt % in H_2_O), hydrogen peroxide solution (30 wt % in H_2_O) was diluted with five (5) parts of deionized water. Deionized water was produced using a Mill-Q A10 system (Millipore, Billerica, MA, USA). The ferric and ferrous iron content of the particle dispersions was colorimetrically determined using the phenanthroline method [101].

### Preparation of multicore particles

For synthesis of MCP (MCP 1 and MCP 2), Fe(II)chloride tetrahydrate was dissolved in deionized water under an air atmosphere, and potassium hydroxide and hydrogen peroxide were successively added under stirring. The resulting MCP were washed with water by magnetic separation, and carboxymethyl dextran sodium salt (CMD-Na) was added and solved under stirring. The mixture was diluted with water and heated at 90°C for 7.5–8 h. Thereafter, several magnetic separation steps were performed to remove the sediment, and the supernatants were combined and washed with water via ultrafiltration and concentrated. The resulting aqueous dispersions were divided into fractions by magnetic separation using water and occasionally KOH solution to obtain the final MCP. For in vivo use, the MCP were concentrated by centrifugation with centrifugal filter units. To the resulting dispersion, D-mannitol and optionally aqueous sodium lactate were added to adjust the pH of the dispersion to a range of 6.5 to 7.5, followed by sterile filtration (syringe filter) and autoclaving. (For details of the reaction, see S1 Protocol).

### Nanoparticle characterization

Nanoparticle size and morphology were analyzed by high-resolution transmission electron microscopy (HRTEM) using a TECNAI G2 20 S-Twin (FEI-Company, Hillsboro OR, USA).

Average core/multicore diameters (d_v_) and size distributions were calculated for each nanoparticle sample by averaging 200 particles from the TEM images using ImageJ software (developed by the National Institutes of Health, Bethesda, Maryland, USA). The hydrodynamic diameters of the MNP were determined by dynamic light scattering (DLS, also referred to as photoelectron correlation spectroscopy, PCS) on a Zetasizer Nano ZS particle analyzer (Malvern Instruments, Worcestershire, UK). For Zetasizer measurement, MNP dispersions were diluted with water to a final concentration of 1 mmol Fe/l. T_1_- and T_2_-relaxivities were measured with a Minispec MQ 40 Time-Domain Nuclear magnetic resonance (TD-NMR) spectrometer at 40°C, 40 MHz and 0.94 T (Bruker, Karlsruhe, Germany). Relaxation coefficients r2 were determined by linear fitting of R2-relaxation rates in relation to iron concentrations.

### Ultrafiltration of nanoparticles

Ultrafiltration was performed using Vivaflow 200 filters with a 100 kDa regenerated cellulose (RC) membrane (Sartorius AG, Göttingen, Germany).

### MPS and M(H) characterization of nanoparticles

MPS characterization of MCP was performed with undiluted samples in a magnetic particle spectrometer (MPS) (Bruker Biospin, Germany) at 10 mT, 25.2525 kHz and 37°C for 10 s. For comparison, Resovist^®^ was diluted with water to give 100 mmol Fe/l and measured under the same conditions. For measurement the samples were filled in Life Technologies polymerase chain reaction (PCR) tubes with sample volumes of 30 **μ**l. The amplitude of the magnetic moment, A_k_, was normalized to the iron content of each sample and is given in Am^2^/mol Fe. M(H) measurements were performed in a 75 **μ**l sample filled in a polycarbonate capsule. The magnetic moment of each sample was measured while increasing the applied magnetic field from 0 to 5 T using an MPMS (Magnetic Property Measurement System, Quantum Design, USA). The background signal caused by empty capsules, diamagnetic susceptibility of the dispersion medium, and demineralized water, was subtracted from the signal obtained for the samples. The magnetization curve was obtained by normalizing the magnetic moment of the sample to its iron content.

### *In vitro* experiments

#### Cellular uptake of MNP

*In vitro* cellular uptake of MCP 1 was tested with nonphagocytic primary mesenchymal stem cells (MSC) from murine (C57BL/6) BM (Thermo Fisher Scientific, Waltham, MA, USA) and a phagocytic murine leukemic macrophage cell line (RAW 264.7) (ATCC Cell Biology Collection, Manassas, Virginia USA). Cells were maintained for up to ten passages as suggested by providers. All in vitro experiments compared cells labeled with MCP 1 or Resovist^®^ vs unlabeled cells_._ The protocols used to obtained intracellular labeled MSC were previously published by our group [21], and similar protocols were used for RAW 264.7 macrophages for comparison. In short, cells were transferred into 6-well plates (4,000 cells/cm^2^), followed by overnight cell synchronization in growth medium. Cells were labeled with MCP 1 or Resovist^®^ using MNP loading concentrations of 0.2 mM or 1 mM with (MCP 1:TA or Resovist^®^:TA) or without protamine sulfate as cationic transfection agent (TA). Cells were incubated with MNP for 24h in corresponding cell culture medium with 1% FBS for culture synchronization [102]. MNP incubation with cells was followed by three washing steps using phosphate-buffered saline (PBS) and collected for iron stain (S6 Fig and S7 Fig) or cell passage into a new 6-well plate (4000 cells/cm^2^). This last step was included to completely remove extracellular MNP and MNP adherent to cell culture plastic material [21]. Cell pellets were collected after removal of extracellular MNP and MNP-intracellular uptake was confirmed by iron staining using a Prussian blue protocol (S8 Fig). Replicates (n = 3) from cell pellets equally treated were used for Fe quantification of intracellular MNP by the colorimetric phenanthroline method described somewhere else [103,104] with some modifications for cell pellets and read at 510 nm. Detailed protocol for cell pellets haven been previously described in our group [21]. The mean MNP uptake was calculated from different experiments (n = 3). Fe concentration was calculated using a standard curve from iron standards with 0, 1, 2, 4, 6, 8, 10, 14 and 18 mg Fe /mL. The mean MNP uptake was normalized to cell number and is reported for MSC (S4 Fig) and for macrophages (S5 Fig).

#### Effect of MCP 1 uptake on cell proliferation

Population doubling time (PDT) was assessed as described elsewhere [21,105]. In short, labeled and unlabeled MSC and RAW 264.7 (S9 Fig) were plated into six-well plates at 2,000 cells per well with complete growth medium and the medium was exchanged every 2 days. The cell population was quantified every 2 days for up to 12 days by automatic cell counting with the CASY Model TT. The following formula was used to calculate the PDT: PDT = T x ln2/ln(N_t_/N_0_), where N_0_ = initial cell number, N_t_ = final cell number, and T = time interval.

### *In vivo* MRI and MPI experiments

Rats were maintained in Type IV Macrolon® cages (Zoonlab, Castrop-Brauxel, Germany) on softwood granulate (Lignocel, J. Rettenmaier, Rosenberg, Germany) under a constant 12-h day/night cycle, a temperature of 21 ± 1°C, and 50 ± 5% relative humidity according to the recommendation *2007/526*/*EC* of the European Commission. Animals received commercial standard pellet feed (ssniff, R-M-H, Soest, Germany) and tap water ad libitum. In vivo experiments in rats were conducted in accordance with the requirements and guidelines of EU directive 2010/63/EU and the German Animal Protection Act. The experiments were approved by the local animal protection committee of the LAGeSo Berlin, Germany. Male rats of the Sprague Dawley^®^ Rat strain (Charles River Laboratories, Sulzfeld, Germany) with a body weight of 300±25 g were examined. Rats were anesthetized prior to and during the imaging procedure using 1.0%–2.5% isoflurane. First MRI examinations were performed on a 1.5 Tesla whole body MRI scanner (Magnetom Sonata; Siemens, Erlangen, Germany) using a commercially available extremity coil. The rats were placed supine on a Styrofoam support and positioned in the center of the coil. Dynamic imaging was performed using a T1-weighted 3D gradient-echo (GRE) fast low-angle shot sequence 2 min after administration. The MNP dispersion was injected into a lateral tail vein as a bolus over 2 seconds. In vivo MPI experiments were performed on a preclinical MPI scanner Bruker 25/20 (Bruker Biospin GmbH, Ettlingen, Germany) at Charité. The standard MPI system 25/20 implements dual-purpose coils to generate the drive-field (DF) for excitation of the nanoparticle dispersion and to receive the induced voltage signals from the magnetization of these MNP simultaneously. In addition a prototype of a separate coil to receive only was manufactured and installed by Bruker in the MPI system at Charité in the x-axis channel to gain up the signal-to-noise-ratio (SNR) [106]. In the MPI measurement, we applied a DF amplitude of 12 mT in all three directions and a selection field gradient of (Gx/Gy/Gz) = (1.25/1.25/2.5) T/m. Following acquisition, images were reconstructed with a matrix of 32x32x16 and a field of view (FOV) of 28x28x14 mm^3^. We have applied a moving average of 5 to the measurement data. The reconstruction is made with the same number of frequency components. Overall 1696 frequency components/equations were selected for both particle systems by choosing the SNR-threshold to 20.77 for MCP 2–2 and 8 for Resovist^®^. In the reconstruction, we have used the Kaczmarz’s algorithm [107] with 5 iterations and a regularization factor of 10^−5^. The nanoparticle dispersion was injected as a bolus into a tail vein, and MPI acquisition started approx. 1 min before injection. MRI examinations for overlaying MPI and MRI images were performed after corresponding MPI experiments on a 1 Tesla ICON small animal MRI scanner (Bruker Biospin GmbH, Ettlingen, Germany) and a T1-weighted 2D gradient-echo (GRE) fast low angle shot sequence in coronary direction was used. For both, the MPI and MRI examinations a compatible small animal carrier (Bruker Biospin GmbH, Ettlingen, Germany) was used.

## Supporting information

S1 Fig TEM images of MCP 1 and MCP 2–2. (PDF)

S2 Fig DLS size distributions (intensity) of MCP. (PDF)

S3 Fig HRTEM images for structural considerations. (PDF)

S4 Fig MCP 1 uptake by MSC. (PDF)

S5 Fig MCP 1 uptake by macrophages. (PDF)

S6 Fig Prussian blue stain for mesenchymal stromal cells (MSC) labeled with multicore particles (MCP 1) and Resovist. (PDF)

S7 Fig Prussian blue stain for macrophages cell line (RAW 264.7) labeled with multicore particles (MCP 1) and Resovist. (PDF)

S8 Fig Prussian blue stain for mesenchymal stromal cells labeled with multicore particles (MCP 1). (PDF)

S9 Fig Population doubling time (PDT). (PDF)

S1 Protocol Nanoparticle synthesis and formulation procedure of MCP 1 and MCP 2. (PDF)
