# Supplementary figures and images for: Novel magnetic multicore nanoparticles designed for MPI and other biomedical applications: From synthesis to first in vivo studies
Source: PLoS One. 2018 Jan 4;13(1):e0190214. doi: 10.1371/journal.pone.0190214 (PMC5754082; doi:10.1371/journal.pone.0190214)

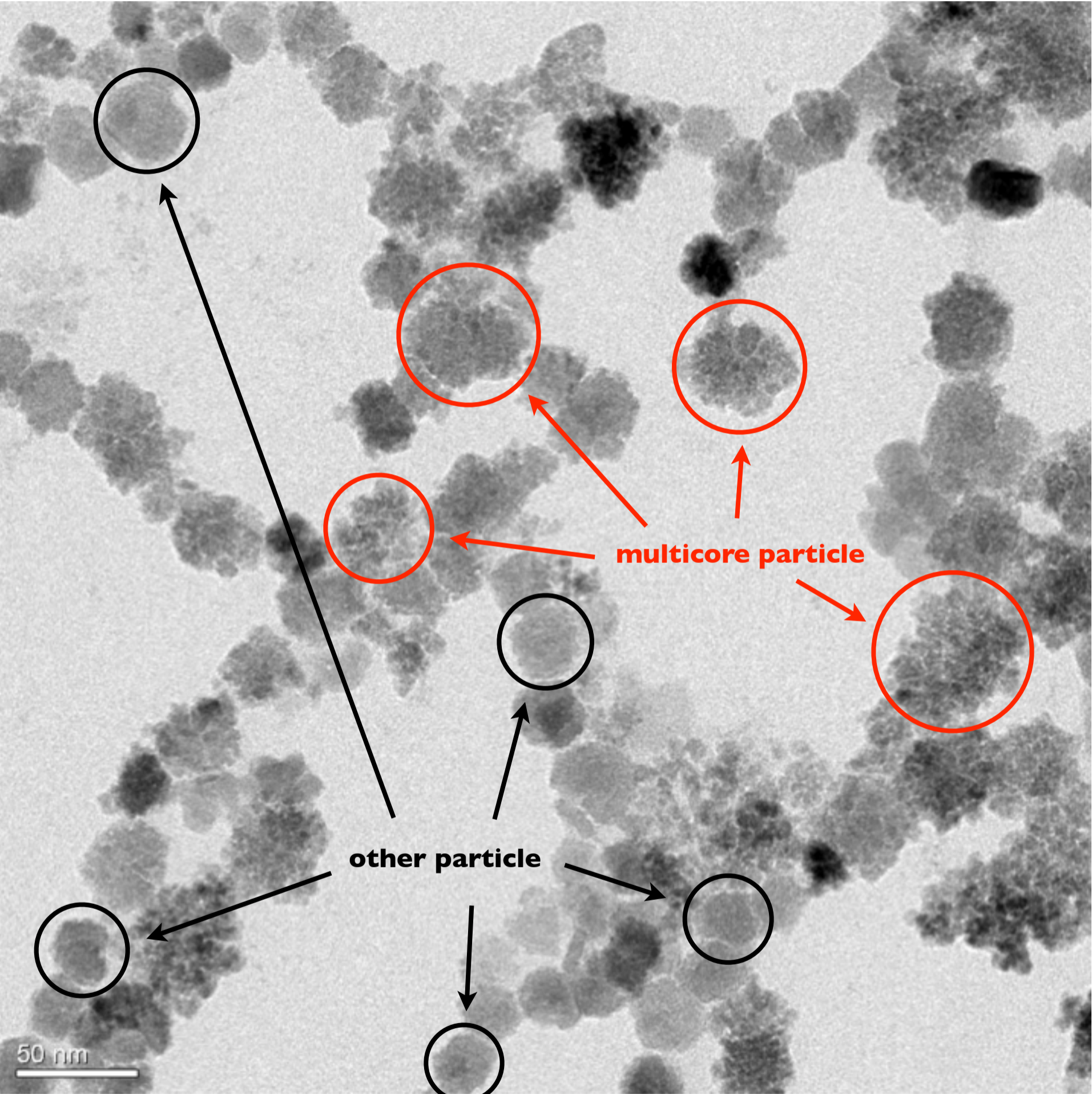

TEM image of MCP 1

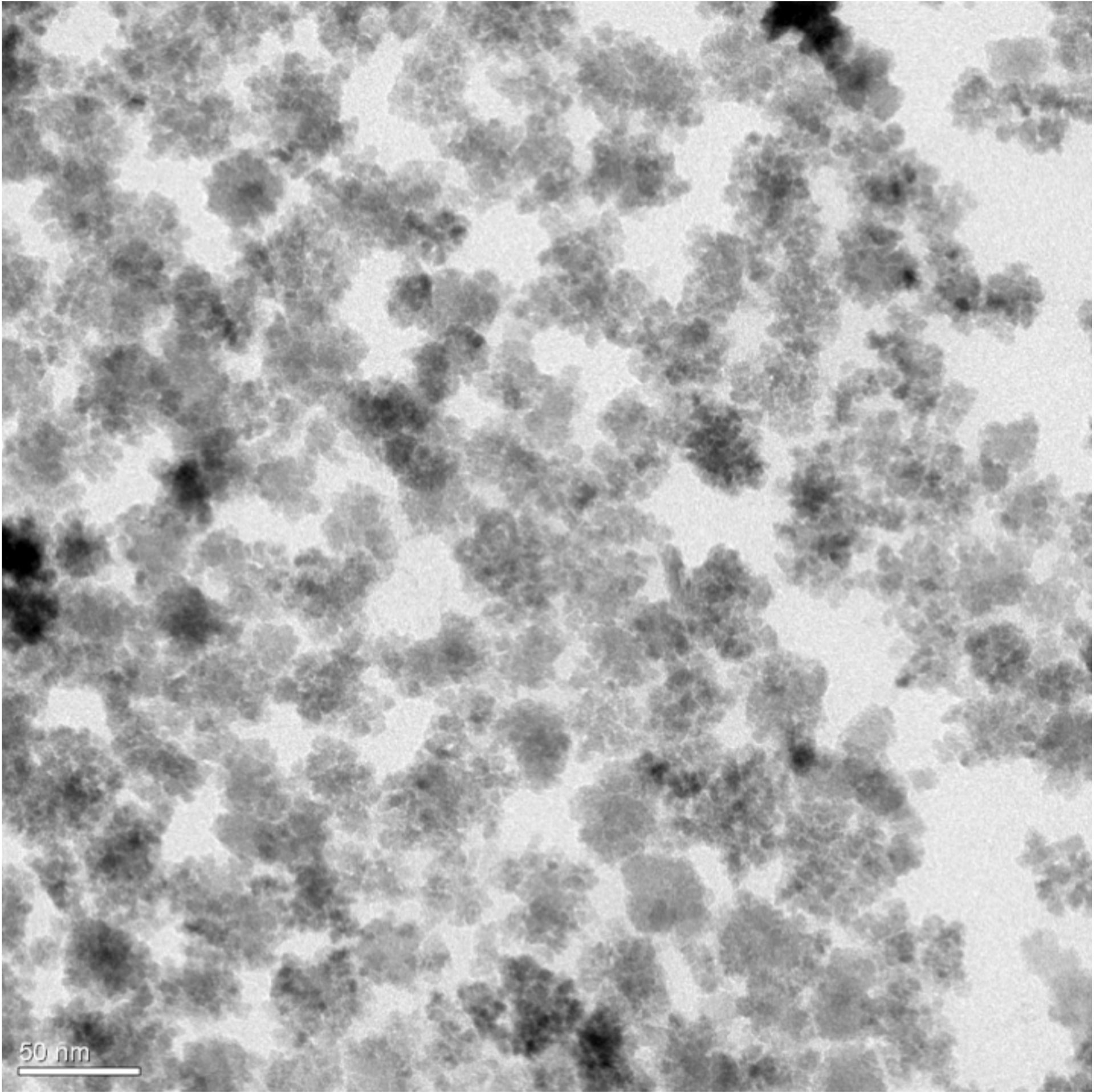

TEM image of MCP 2-2

Supplement: S1 Fig — (PDF) [file pone.0190214.s001.pdf]
