# Supplementary material for: Novel magnetic multicore nanoparticles designed for MPI and other biomedical applications: From synthesis to first in vivo studies
Source: PLoS One. 2018 Jan 4;13(1):e0190214. doi: 10.1371/journal.pone.0190214 (PMC5754082; doi:10.1371/journal.pone.0190214)
Supplement: S3 Fig — (PDF) [file pone.0190214.s003.pdf]

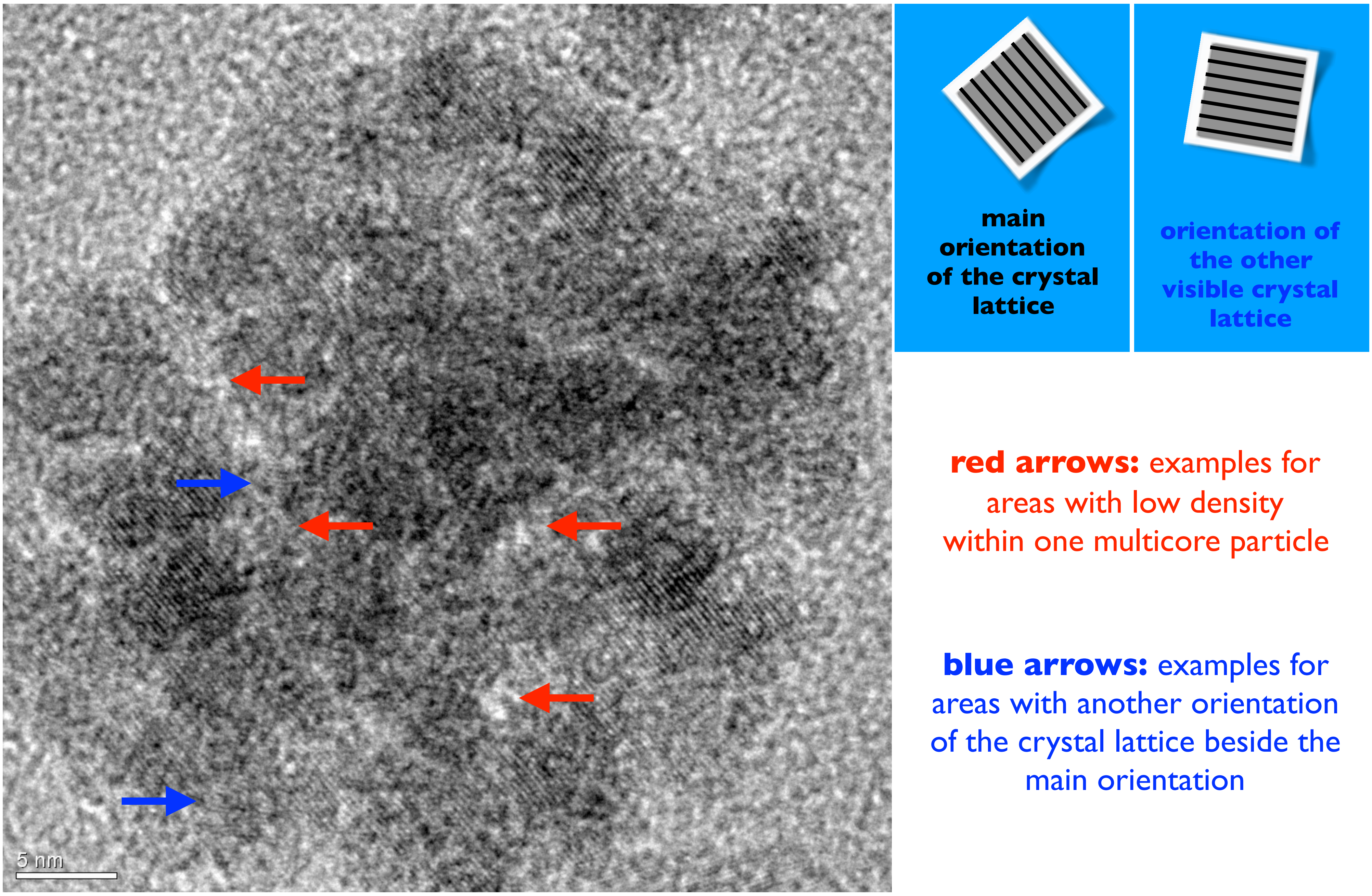
5 nm
main orientation of the crystal lattice
orientation of the other visible crystal lattice
red arrows: examples for areas with low density within one multicore particle
blue arrows: examples for areas with another orientation of the crystal lattice beside the main orientation

MCP 1

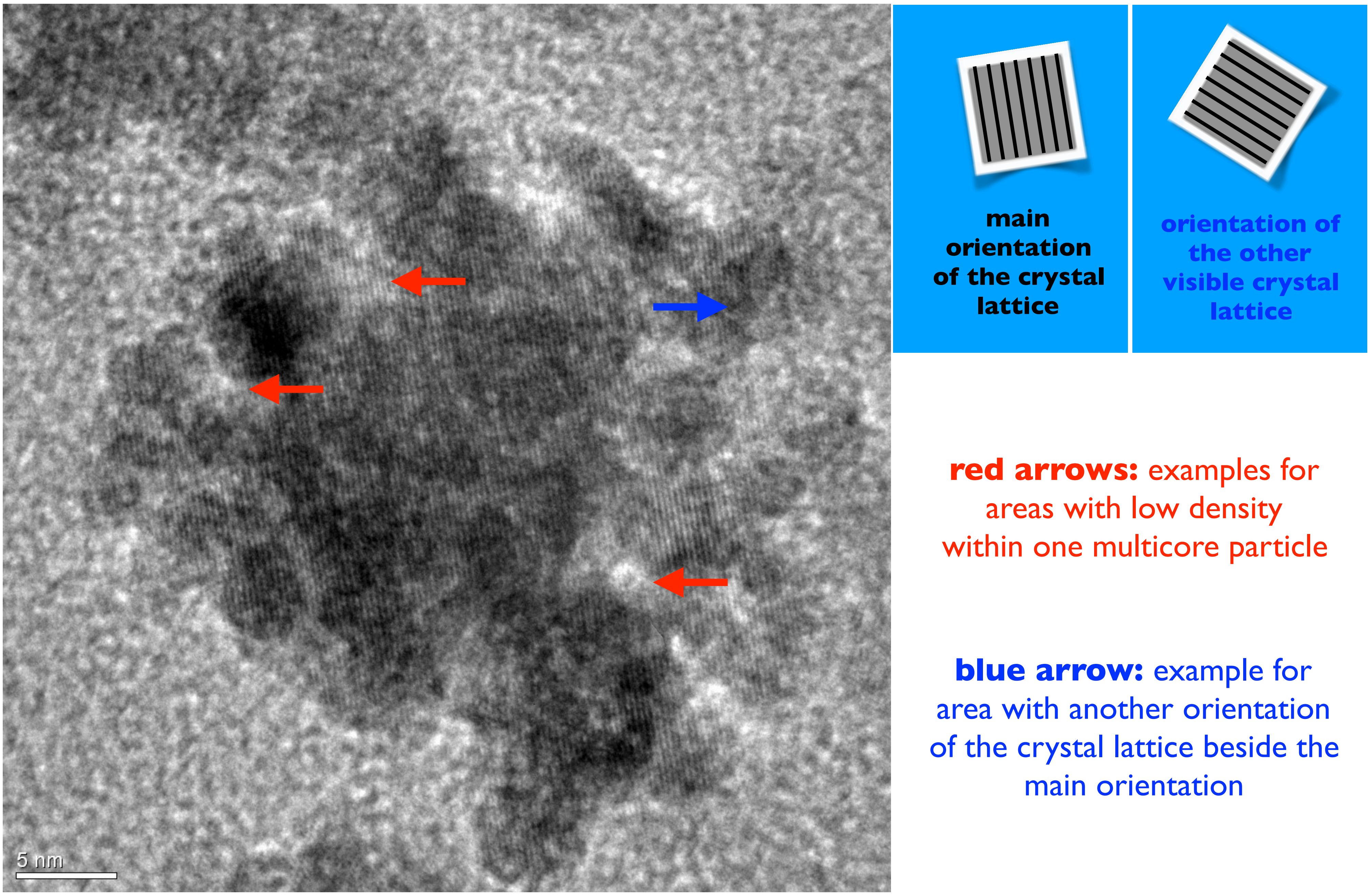
5 nm
main orientation of the crystal lattice
orientation of the other visible crystal lattice
red arrows: examples for areas with low density within one multicore particle
blue arrow: example for area with another orientation of the crystal lattice beside the main orientation

MCP 1

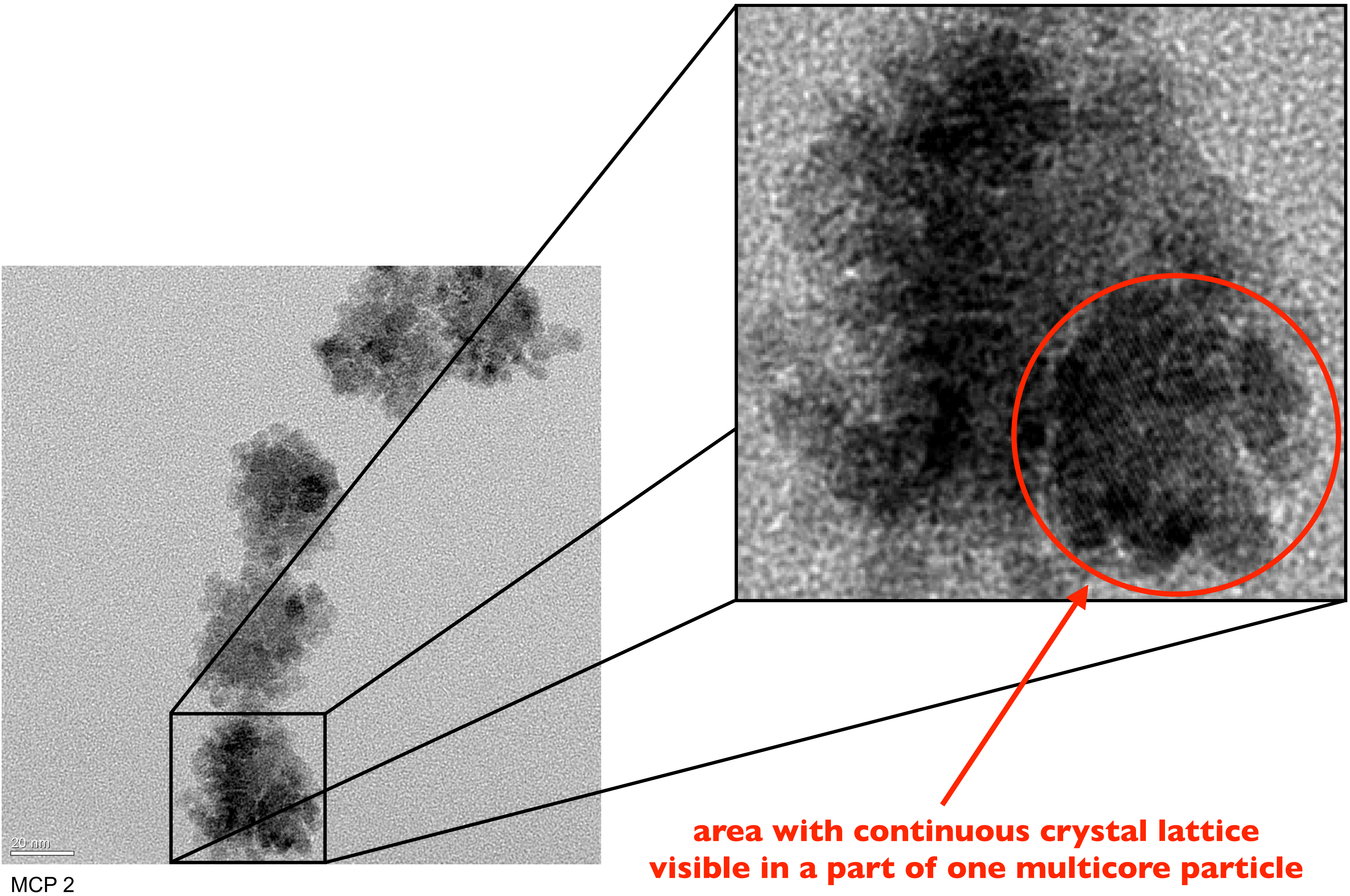
20 nm
area with continuous crystal lattice
visible in a part of one multicore particle

MCP 2
