# Supplementary material for: Novel magnetic multicore nanoparticles designed for MPI and other biomedical applications: From synthesis to first in vivo studies
Source: PLoS One. 2018 Jan 4;13(1):e0190214. doi: 10.1371/journal.pone.0190214 (PMC5754082; doi:10.1371/journal.pone.0190214)
Supplement: S4 Fig — (PDF) [file pone.0190214.s004.pdf]

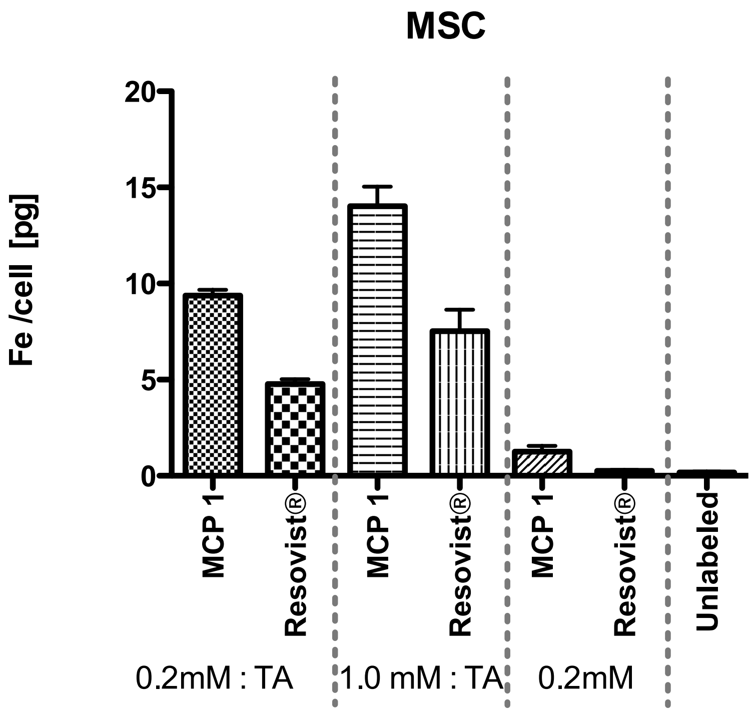

MCP 1 uptake by MSC: Mesenchymal stem cells (MSC) uptake more efficiently MCP 1 than Resovist® nanoparticles. Uptake of both nanoparticles was increased by increasing nanoparticle loading concentration (0.2mM to 1mM Fe) under assistance of protamine sulfate, a cationic transfection agent (TA). The highest label efficiency was achieved after 24h incubation with MCP 1: TA Protamine (1mM: 48μg/mL) followed by incubation with MCP 1 : TA 0.2mM : 12μg/mL .
