# Supplementary material for: Novel magnetic multicore nanoparticles designed for MPI and other biomedical applications: From synthesis to first in vivo studies
Source: PLoS One. 2018 Jan 4;13(1):e0190214. doi: 10.1371/journal.pone.0190214 (PMC5754082; doi:10.1371/journal.pone.0190214)
Supplement: S5 Fig — (PDF) [file pone.0190214.s005.pdf]

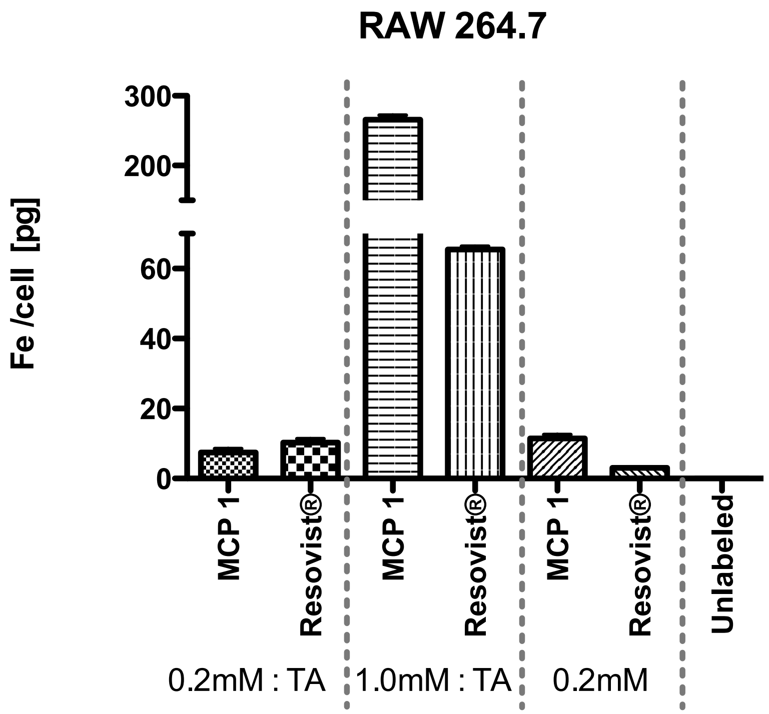

MCP 1 uptake by Macrophages: Intracellular labeling of mouse leukemic macrophage cell line (RAW 264.7) is achieved by phagocytosis of MCP 1 without transfection agent (TA). The use of MNP: TA (0.2mM : TA) complexes did not increase significantly the total uptake of MCP 1 or Resovist®, but significant increase was achieved after cell incubation with higher amount of MNP (1.0mM: TA).
