# Supplementary material for: Novel magnetic multicore nanoparticles designed for MPI and other biomedical applications: From synthesis to first in vivo studies
Source: PLoS One. 2018 Jan 4;13(1):e0190214. doi: 10.1371/journal.pone.0190214 (PMC5754082; doi:10.1371/journal.pone.0190214)
Supplement: S6 Fig — (PDF) [file pone.0190214.s006.pdf]

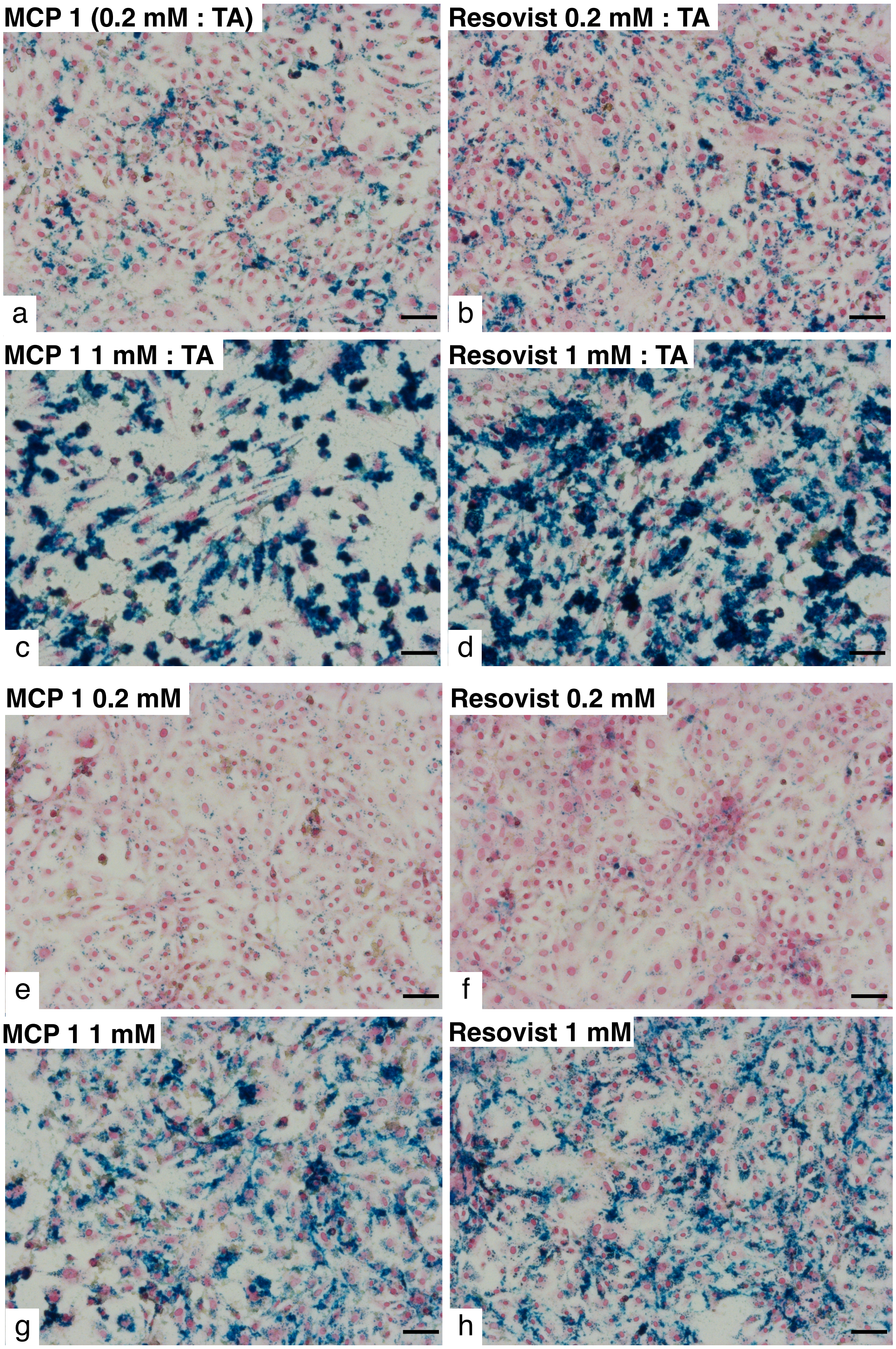

Prussian blue stain for Mesenchymal stromal cells (MSC) labeled with multicore particles (MCP 1) and Resovist. Iron stain for MSC incubated during 24h for MNP-loading concentrations (0.2mM and 1mM) with or without protamine sulfate as transfection agent (TA). After 24h incubation, increased positive iron stain associated with MSC was observed when incubating with TA (a to d) vs without TA (e to h) and at increasing MNP-loading concentration (g, h). However, both conditions, induced MNP aggregation in culture plates (c, d and g, h). This shows higher amount of extracellular MCP 1 and Resovist after 24h incubation protocols. Scale bar corresponds to 500μm.
