# Supplementary material for: Novel magnetic multicore nanoparticles designed for MPI and other biomedical applications: From synthesis to first in vivo studies
Source: PLoS One. 2018 Jan 4;13(1):e0190214. doi: 10.1371/journal.pone.0190214 (PMC5754082; doi:10.1371/journal.pone.0190214)
Supplement: S7 Fig — (PDF) [file pone.0190214.s007.pdf]

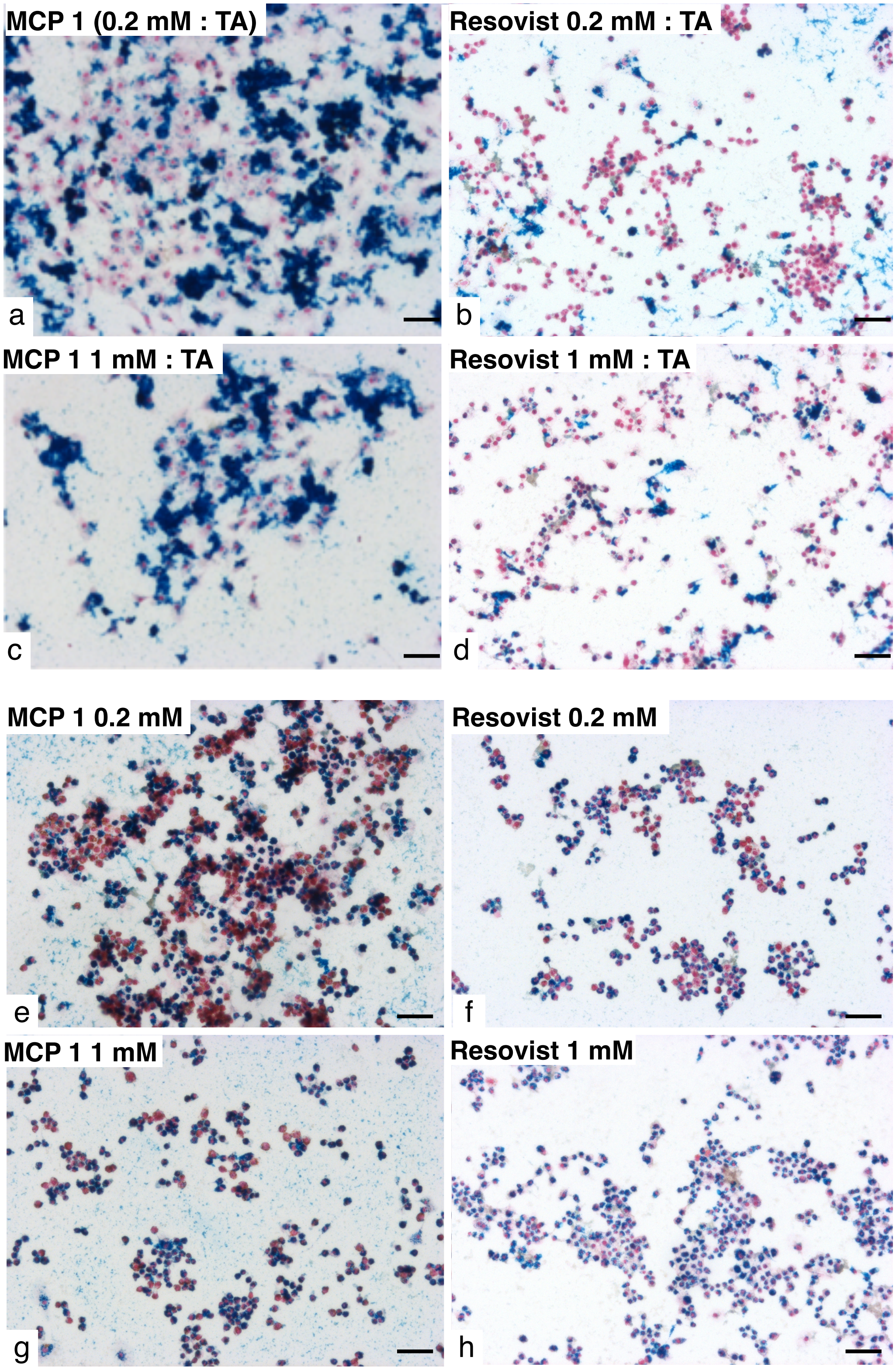

Prussian blue stain for Macrophages cell line (RAW 264.7) labeled with multicore particles (MCP 1) and Resovist. Iron stain for macrophages incubated during 24h for MNP-loading concentrations (0.2mM and 1mM) with or without protamine sulfate as transfection agent (TA). After 24h incubation, High aggregation of MCP 1 was observed when incubated with TA (a and c). These images suggest better uptake of MCP 1 and Resovist by macrophages without using TA (e to h). Scale bar corresponds to 500μm.
