# Supplementary material for: Novel magnetic multicore nanoparticles designed for MPI and other biomedical applications: From synthesis to first in vivo studies
Source: PLoS One. 2018 Jan 4;13(1):e0190214. doi: 10.1371/journal.pone.0190214 (PMC5754082; doi:10.1371/journal.pone.0190214)
Supplement: S8 Fig — (PDF) [file pone.0190214.s008.pdf]

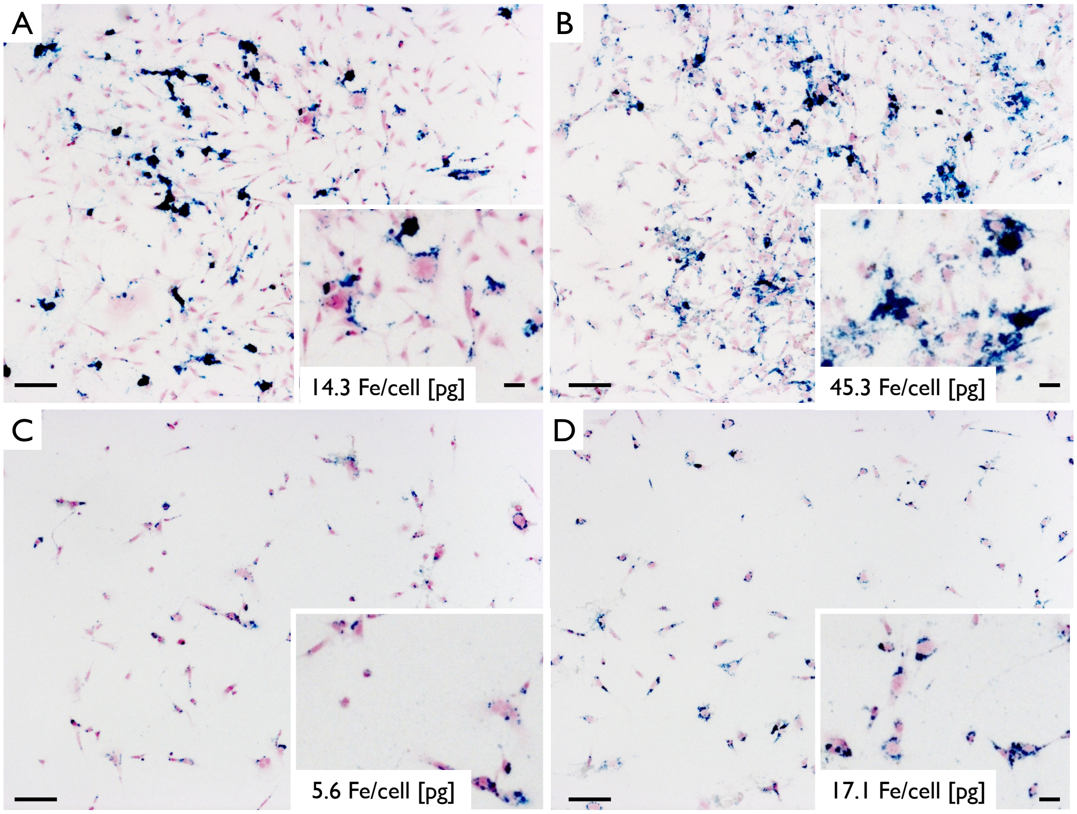

Prussian blue stain for Mesenchymal stromal cells labeled with multicore particles (MCP 1). Iron stain of MSC incubated during 24h incubation with MCP 1 and additional extracellular matrix disruption to remove extracellular MCP 1. MSC incubated with MCP 1 at 0.2mM : Protamine sulfate (A) or 1mM without TA (B) results in positive iron stain associated to cells. However, high amount of extracellular iron is visible after both treatments (A and B). Additional extracellular matrix disruption and passage, reduced extracellular and thereby unspecific iron quantification (C and D). Scale bar corresponds to 500μm.
