# Supplementary material for: Novel magnetic multicore nanoparticles designed for MPI and other biomedical applications: From synthesis to first in vivo studies
Source: PLoS One. 2018 Jan 4;13(1):e0190214. doi: 10.1371/journal.pone.0190214 (PMC5754082; doi:10.1371/journal.pone.0190214)
Supplement: S9 Fig — (PDF) [file pone.0190214.s009.pdf]

A

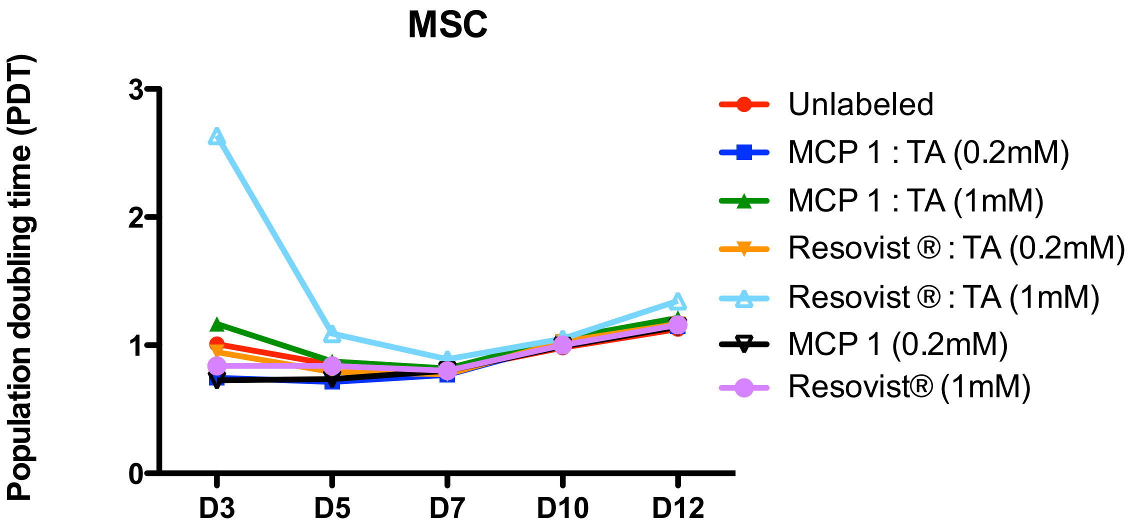

B

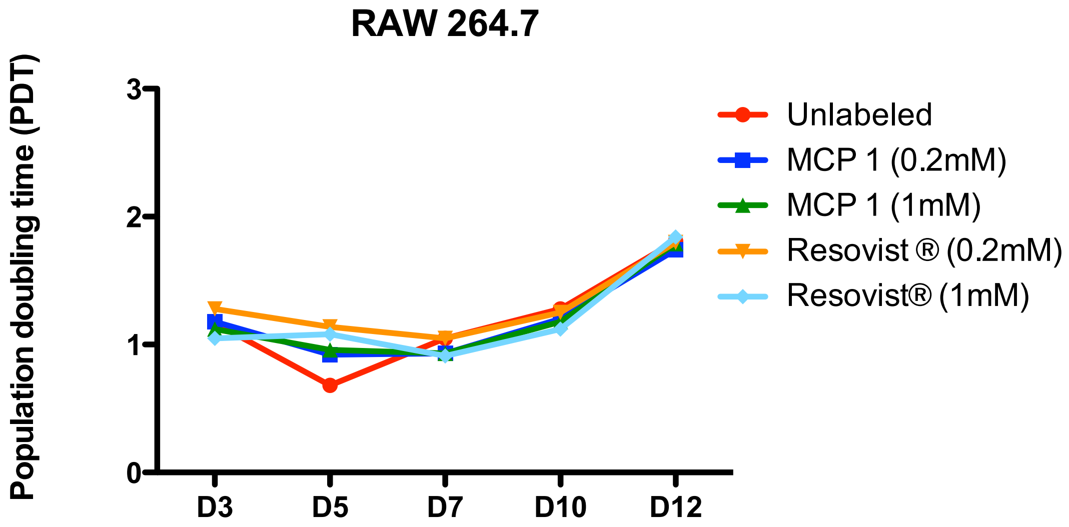

Population doubling time (PDT): A) MSC and B) Macrophages labeled with MCP 1 maintained similar PDT in comparison with Resovist® labeled and unlabeled cells during 12 days. PDT (PDT= T x ln2 /ln (A/A0): T= time between cell counts, A= final cell number , A0= initial cell number).
