# Supplementary material for: Novel magnetic multicore nanoparticles designed for MPI and other biomedical applications: From synthesis to first in vivo studies
Source: PLoS One. 2018 Jan 4;13(1):e0190214. doi: 10.1371/journal.pone.0190214 (PMC5754082; doi:10.1371/journal.pone.0190214)
Supplement: S1 Protocol — (PDF) [file pone.0190214.s010.pdf]

Synthesis of MCP 1

3,96g (19.92 mmol) Fe(II)chloride tetrahydrate was dissolved in 200 ml of deionized water under an air atmosphere (23wt% oxygen) and stirred for 5 min at room temperature. Thereafter, 44 ml of potassium hydroxide (1N) was added. After stirring for 10 min, 2 ml of hydrogen peroxide (5% in water) was added and the dispersion was stirred again for 10 min. Subsequently, magnetic separation was realized for 12 min, the supernatant was decanted and rejected. The sediment was mixed up in 200 ml of deionized water and placed on a magnet for another 15 min. After that 8 g of carboxymethyl dextran sodium salt (CMD-Na) was added and the dispersion was stirred for 10 min at room temperature. The mixture was diluted with water to 250 ml and heated at 90°C for 450 min. Thereafter magnetic separation was performed for 23 min, the supernatant was decanted and the sediment suspended in 165 ml of water and subjected to another magnetic separation for 23 min. This process (magnetic separation, removing of the supernatant and following resuspension with water) was repeated until the resulting supernatant is turbid. The supernatants were combined and washed with water via ultrafiltration using a Vivaflow 200 filter (100 kDa RC) until the filtrate has a conductivity value of less than 10 µS and concentrated to about 67 ml. The dispersion is placed on a magnet for 15 min, and about 60 ml is removed by pipette (supernatant 1), the sediment (sediment 1) is preserved and supernatant 1 placed on a magnet overnight, and about 45 ml (dispersion 1) is pipetted off to obtain dispersion 1. The sediment 1 is mixed up with 67 ml of water and 1N KOH solution was added drop-wise until the pH of the dispersion is about 10. Following magnetic separation for 15 min, about 80 ml of dispersion is pipetted off (supernatant 2) and is placed on a magnet overnight, and about 70 ml is pipetted off to get dispersion 2 (**MCP 1**).

Synthesis of MCP 2

3,96g (19.92 mmol) Fe(II)chloride tetrahydrate was dissolved in 200 ml of deionized water under an air atmosphere (23wt% oxygen) and stirred for 5 min at room temperature. Thereafter, 44 ml of potassium hydroxide (1N) was added. After stirring for 10 min, 2 ml of hydrogen peroxide (5% in water) was added and the dispersion was stirred for 10 min, then 1 ml of hydrogen peroxide (5% in water) was added and the dispersion was stirred again for another 10 min. Subsequently, magnetic separation was realized for 15 min, the supernatant was decanted and rejected. The sediment was mixed up with 100 ml of deionized water. After that 8 g of carboxymethyl dextran sodium salt (CMD-Na) was added

under stirring and stirred for 10 min at room temperature. The mixture (about 190 ml) was heated at 90°C for 480 min. Thereafter magnetic separation was performed for 23 min, the supernatant was decanted and the sediment suspended in 200 ml of water and subjected to another magnetic separation for 23 min. This process (magnetic separation, removing of the supernatant and following resuspension with water) was repeated until the resulting supernatant is turbid. The supernatants were combined and washed with water via ultrafiltration using a Vivaflow 200 filter (100 kDa RC) until the filtrate has a conductivity value of less than 10 µS and concentrated to about 40 ml. The dispersion was placed on a magnet for 72 h, and about 30 ml was removed by pipette (dispersion 1), the sediment was mixed up with 25 ml deionized water, the pH of the dispersion was adjusted to a value of 10 by 1N potassium hydroxide solution and placed on a magnet overnight. The next day the supernatant was removed by pipette (dispersion 2) and the sediment was mixed up with 25 ml deionized water. The pH of the dispersion was adjusted to a value of 10 by 1N potassium hydroxide solution and placed again on a magnet overnight. The supernatant was removed by pipette (dispersion 3) and the sediment was mixed up with 25 ml deionized water and placed on a magnet overnight, the supernatant (dispersion 4, **MCP 2-1**) removed by pipette. The sediment was mixed up with 25 ml deionized water, placed again on a magnet overnight and the supernatant (dispersion 5, **MCP 2-2**) is removed by pipette.

Formulation of MCP 1 and MCP 2 for in vivo use

MNP was concentrated by centrifugation with 3112 x g using Amicon Ultra-15 Centrifugal Filter Units (PLHK Ultracel-PL Membrane, 100 kDa). To the concentrated dispersions an amount of D-mannitol is added to yield an 6% w/w D-mannitol solution. The pH of the dispersion should be in an range from 6.5 to 7.5. If the dispersion is in the acid range, pH was adjusted with aqueous sodium lactate solution (200 g/l; 1.78 M)). Thereafter the dispersion was passed through 0,2 µm (cellulose acetate) syringe filter (sterile filtration) and autoclaved at 120°C, 1 bar for 20 min.
